# Clinical Decision Support Using Speech Signal Analysis: Systematic Scoping Review of Neurological Disorders

**DOI:** 10.2196/63004

**Published:** 2025-01-13

**Authors:** Upeka De Silva, Samaneh Madanian, Sharon Olsen, John Michael Templeton, Christian Poellabauer, Sandra L Schneider, Ajit Narayanan, Rahmina Rubaiat

**Affiliations:** 1 Department of Computer Science and Software Engineering Auckland University of Technology Auckland New Zealand; 2 Rehabilitation Innovation Centre Auckland University of Technology Auckland New Zealand; 3 School of Computer Science and Engineering University of South Florida Tampa, FL United States; 4 School of Computing and Information Sciences Florida International University Miami, FL United States; 5 Department of Communicative Sciences & Disorders St Mary’s College Notre Dame, IN United States; 6 Knight Foundation of Computing & Information Sciences Florida International University Miami, FL United States

**Keywords:** digital health, health informatics, digital biomarker, speech analytics, artificial intelligence, machine learning

## Abstract

**Background:**

Digital biomarkers are increasingly used in clinical decision support for various health conditions. Speech features as digital biomarkers can offer insights into underlying physiological processes due to the complexity of speech production. This process involves respiration, phonation, articulation, and resonance, all of which rely on specific motor systems for the preparation and execution of speech. Deficits in any of these systems can cause changes in speech signal patterns. Increasing efforts are being made to develop speech-based clinical decision support systems.

**Objective:**

This systematic scoping review investigated the technological revolution and recent digital clinical speech signal analysis trends to understand the key concepts and research processes from clinical and technical perspectives.

**Methods:**

A systematic scoping review was undertaken in 6 databases guided by a set of research questions. Articles that focused on speech signal analysis for clinical decision-making were identified, and the included studies were analyzed quantitatively. A narrower scope of studies investigating neurological diseases were analyzed using qualitative content analysis.

**Results:**

A total of 389 articles met the initial eligibility criteria, of which 72 (18.5%) that focused on neurological diseases were included in the qualitative analysis. In the included studies, Parkinson disease, Alzheimer disease, and cognitive disorders were the most frequently investigated conditions. The literature explored the potential of speech feature analysis in diagnosis, differentiating between, assessing the severity and monitoring the treatment of neurological conditions. The common speech tasks used were sustained phonations, diadochokinetic tasks, reading tasks, activity-based tasks, picture descriptions, and prompted speech tasks. From these tasks, conventional speech features (such as fundamental frequency, jitter, and shimmer), advanced digital signal processing–based speech features (such as wavelet transformation–based features), and spectrograms in the form of audio images were analyzed. Traditional machine learning and deep learning approaches were used to build predictive models, whereas statistical analysis assessed variable relationships and reliability of speech features. Model evaluations primarily focused on analytical validations. A significant research gap was identified: the need for a structured research process to guide studies toward potential technological intervention in clinical settings. To address this, a research framework was proposed that adapts a design science research methodology to guide research studies systematically.

**Conclusions:**

The findings highlight how data science techniques can enhance speech signal analysis to support clinical decision-making. By combining knowledge from clinical practice, speech science, and data science within a structured research framework, future research may achieve greater clinical relevance.

## Introduction

### Background

Clinical decision-making is key to effective patient care. It fundamentally relies on evidence derived from validated assessment tools. These assessments typically combine subjective observations (ie, observable signs and patient-reported symptoms) and objective measurements (ie, physiological measurements [1-4], such as blood pressure and heart rate) [5]. However, these traditional assessments face inherent challenges due to clinical feature complexity, potential clinician bias, varying levels of expertise, and substantial instrumentation costs.

Digital biomarkers emerge as a transformative paradigm in clinical decision support, providing precise, objective measurements that extend beyond traditional assessments. Through smartphone and wearable sensors, these biomarkers capture granular patient data previously inaccessible to clinicians. For example, motion sensors quantify fine motor control through typing patterns and touch screen interactions, whereas accelerometers and gyroscopes measure gait parameters, including stride length variability, postural sway, and turning speed. Cognitive functions can be continuously assessed through patterns of smart device use, including response times, error rates, and daily activity rhythms [6]. Speech signals captured through recording instruments can detect subtle speech variations such as changes in fundamental frequency, rhythmic disturbances, voice quality, articulatory precision, and prosodic features that may indicate psychiatric conditions [7,8]. Among these emerging digital biomarkers, speech feature measurements provide clinical insights through a noninvasive, nonintrusive approach using low-cost smart and wearable digital devices [9] at scale in real time and offline modes [10,11].

Speech production is a complex task that involves the orchestration and coordination of different body systems [12]. Deficiencies in any component of the speech production system could manifest in speech pattern changes [13]. Therefore, these alterations provide objective, quantifiable markers for differential diagnosis and disease progression monitoring. They can also provide insights into normal and pathological biological processes [6,14]. Traditional clinical speech assessment mostly relies on standardized tests administered by speech-language pathologists to assess motor speech production [13,15,16] for conditions such as traumatic brain injury, stroke [17], Parkinson disease (PD), and multiple sclerosis (MS) [18]. In current practices, acoustic measures and auditory perceptual judgments [19] are typically used based on guidelines such as the Darley, Aronson, and Brown system [20,21] in the characterization of motor speech control deficits. Despite these approaches and guidelines, limitations exist in these conventional procedures. Some assessments are time-consuming, require specialized clinical experts [22,23], and heavily rely on clinicians’ subjective perceptual judgments. This subjectivity introduces interpretation variability [24] and challenges in maintaining consistent interrater reliability [25,26] although there is evidence showing consistent auditory-perceptual assessments [27]. The environmental, physical, and emotional states of patients during the assessments can also lead to further inconsistencies [22].

Therefore, digital speech signal analysis (hereafter referred to as “speech analysis”) offers a promising solution through enhanced objectivity and retest capability [22] with reduced clinical burden and improved accuracy. Opportunities exist in identifying specific speech features or speech patterns related to different health conditions, including neurological diseases [18,28]. Recent advances in artificial intelligence and machine learning (ML) techniques further support detecting subtle changes and fine-grained speech features that can be related to associated health conditions [29].

There is a growing interest in investigating speech as a digital biomarker for clinical decision support. Several studies have explored speech analysis for clinical assessments of specific neurological health conditions. The prosodic aspect of speech production in PD was reviewed in the study by Moro-Velasquez and Dehak [30], whereas Moro-Velasquez et al [31] reviewed the articulatory and phonatory aspects. Early detection of PD using speech features and ML was discussed in the study by Gullapalli and Mittal [32]. Automatic speech assessment in Alzheimer disease (AD) was reviewed in the study by Pulido et al [33], whereas Martínez-Nicolás et al [34] included mild cognitive impairment (MCI) as well. Both the studies by de la Fuente Garcia et al [35] and Petti et al [36] also focused on AD but considered language assessments in addition to speech. Automated speech and language features were reviewed as an indication of deficits in content organization and thought processes considering related neurological impairments such as AD and MCI [15]. A meta-analysis of acoustic features on autism spectrum disorder (ASD) [37] and articulatory impairments in neurodegenerative motor diseases [38] also reviewed and compiled knowledge on speech signal analysis.

### Objectives

Despite the growing body of research on speech analysis for different diseases, the field lacks a comprehensive synthesis of available studies, their approaches, and clinical applications for neurological diseases. Therefore, in this research, we aimed to investigate the technology revolution and trends in speech analysis to understand the key concepts and research processes across different neurological conditions. Our review focused on studies that investigated the physical features of speech, focusing on the underlying digital acoustic features instead of the content. We aimed to review the clinical and technical perspectives of the research process in relation to clinical application. Given the interdisciplinary nature of the research field and the premature stage of clinical integration, we emphasize the importance of establishing a suitable research framework to guide future research. We also proposed a research framework for speech analysis in clinical decision support adapting a design science research process.

## Methods

### Overview

The review process adhered to the PRISMA-ScR (Preferred Reporting Items for Systematic Reviews and Meta-Analyses extension for Scoping Reviews) guidelines [39] (Multimedia Appendix 1). We conducted a systematic scoping review following a predefined protocol to comprehensively identify, evaluate, and synthesize relevant literature. Our review was guided by the following research questions:

- In which health care disciplines or for which health conditions has speech analysis been investigated? (research question 1)
- What types of clinical outcomes or clinical services might be possible using speech analysis? (research question 2)
  What data science methods have been used for these speech analyses? (research question 2.1)

No protocol was registered for this review.

### Sources and Eligibility Criteria

As a part of a larger research program, we conducted a comprehensive data collection. The review was limited to the Scopus, IEEE Xplore, Google Scholar, MEDLINE via PubMed, SpringerLink, and ScienceDirect databases. Textbox 1 lists the inclusion and exclusion criteria followed during article selection.

**Inclusion and exclusion criteria.**

**Inclusion criteria**

- Language: publications in English only
- Publication type: peer-reviewed journal articles and conference proceedings
- Publication period: published between January 2010 and December 2022
- Participants: human participants of any age
- Type of research: primary research
- Study characteristics:
  - Carried out analysis of features of speech signals
  - Used features for analysis that were language independent
  - Used data science approaches to derive clinical insights on health conditions that impact speech (statistical analyses and traditional machine learning and deep learning approaches were considered interesting data science approaches)

**Exclusion criteria**

- Language: non–English-language publications
- Publication type: book chapters, theses, abstracts, editorials, and gray literature
- Publication period: published before January 2010
- Participants: nonhuman participants
- Study characteristics:
  - Analyzed only transcriptions or linguistics features, such as grammar and semantics
  - Investigated speech signal analysis on functional and structural voice disorders
  - Investigated only nonverbal audio sounds, such as breathing, coughing, crying, and snoring
  - Investigated only linguistic features of speech
  - Did not focus on a health care aspect (eg, the studies analyzed speech signals for emotion recognition without concerning a health condition)

### Search Strategy and Study Selection

The following keywords and their synonyms were used in building searching queries and extracting articles: “Speech,” “Analysis,” “Health care,” “Data Science,” and “Artificial Intelligence.” The search strategy for the databases is presented in Multimedia Appendix 2.

The identified articles were exported to a reference management software where duplicates were removed. We then screened articles based on the titles and abstracts, followed by a full-text search. After a detailed examination of the retrieved full texts, those that met the eligibility criteria (N=389) were selected for the quantitative analysis of the research landscape. The full texts of articles that met the defined eligibility criteria and focused on a neurological disease with primary data collection (72/389, 18.5%) were included in this analysis and added to NVivo (Lumivero) [40], a qualitative data analysis software. We used qualitative content analysis followed by a mix of deductive and inductive reasoning [41] to identify key concepts in speech analysis for clinical decision support.

The details of the included research articles can be found in Multimedia Appendix 3 [42-113].

### Study Characteristics

**Figure 1:**
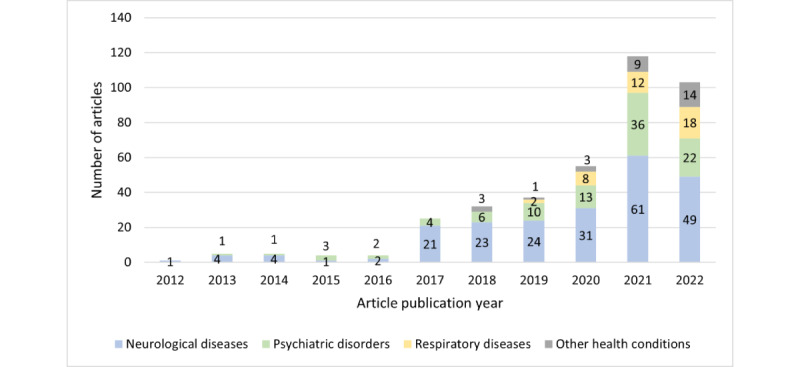
Yearly distribution of the eligible articles (N=389) and their clinical disciplines.

The clinical disciplines and the yearly distribution of the overall eligible articles (N=389) are plotted in Figure 1 to understand the potential health care disciplines for digital clinical speech analysis to answer our research question 1. The evaluation of the research field was clearly evident, with an increasing number of articles over the years. Neurological diseases attracted the highest research interest (221/389, 56.8%), including AD, PD, and amyotrophic lateral sclerosis (ALS). Psychiatric disorders such as depression, bipolar disorder, and schizophrenia were among the next most investigated disease categories (98/389, 25.2%), followed by respiratory diseases (40/389, 10.3%). The remaining articles (30/389, 7.7%) investigated other health conditions, such as heart disease and cancer.

**Figure 2:**
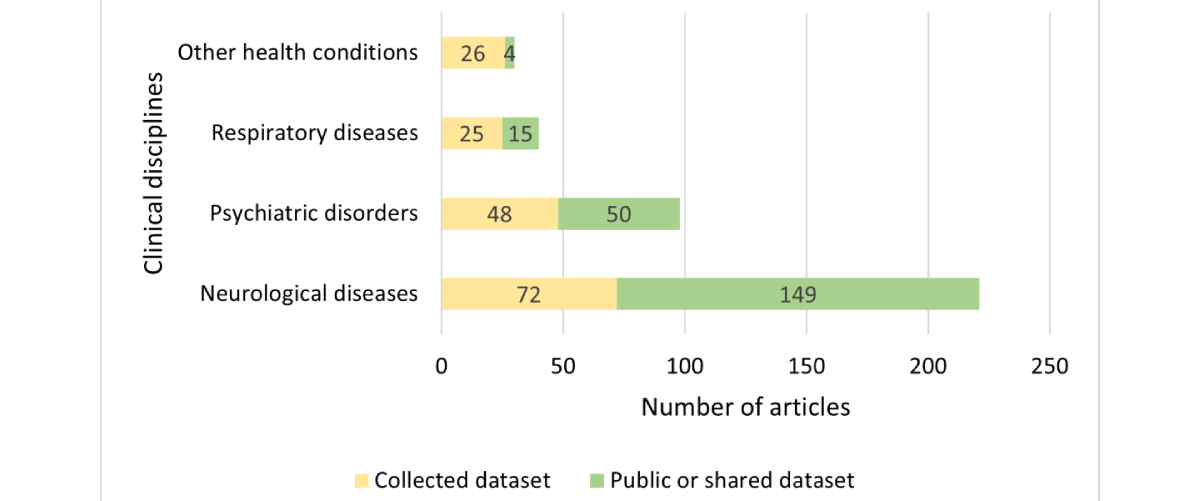
Distribution of the eligible articles (N=389) based on their data sourcing approach.

Data sourcing in the studies varied across public datasets, institutionally shared datasets, and primary data collections (Figure 2). In this review, we focused on studies with primary data collection as they described the full research process from problem formulation, data collection, data analysis, and model building to model evaluation. Therefore, the analysis of this review considered 18.5% (72/389) of the studies, which investigated neurological diseases using primary data collection.

## Results

### Overview

**Figure 3:**
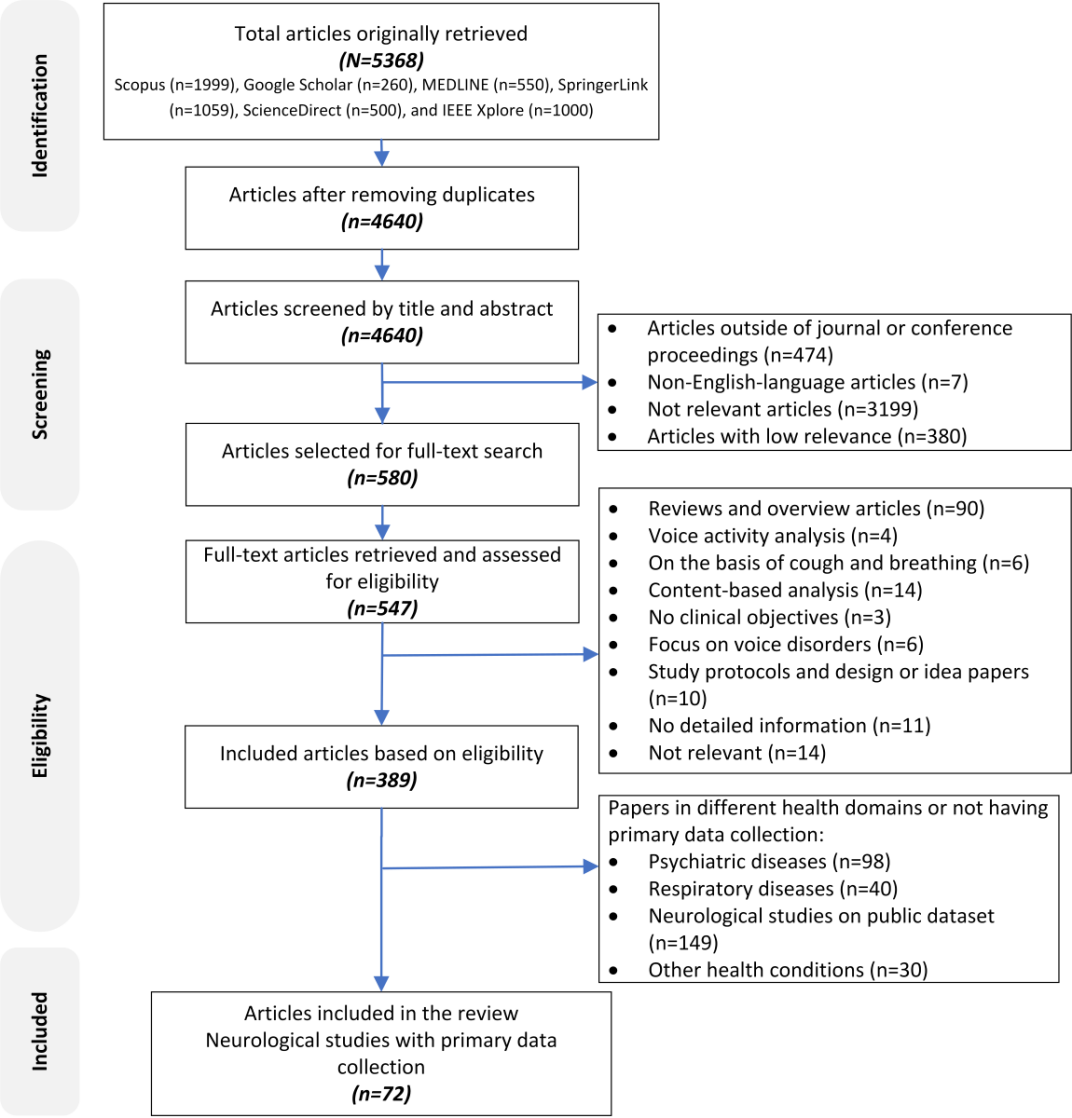
PRISMA (Preferred Reporting Items for Systematic Reviews and Meta-Analyses) flowchart for the study screening and selection process.

Figure 3 presents the detailed overview of the literature search and study selection process for this study. The qualitative content analysis of the 18.5% (72/389) of the studies on neurological diseases revealed the following key themes regarding digital speech signal analysis: (1) health condition and clinical purpose, (2) speech data (speech tasks and speech features), (3) data science approaches and evaluations, and (4) clinical applications. The following subsections provide details on these themes. The details of the included research articles can be found in Multimedia Appendix 3 [42-113].

### Neurological Conditions and Clinical Purposes

#### Overview

Many neurological conditions affect the sensorimotor control of speech movements (eg, PD) or cognitive processes, specifically memory and language and perceptual processing (eg, AD and MCI). While thought formulation and motor planning are distinct in speech production [114], alterations in motor planning can occur alongside cognitive impairments, as discussed in various stages of AD [115]. This can cause acoustic changes in the physical speech signal.

Among the neurological diseases, PD (40/72, 56%) was the most investigated disorder, followed by AD and cognitive impairment (12/72, 17%). Other investigated disorders were MS (5/72, 7%), ALS (4/72, 6%), mild traumatic brain injury (mTBI; 3/72, 4%), Huntington disease (HD; 2/72, 3%), and ASD (2/72, 3%). A total of 4 articles focused on clinical purposes related to apathy (n=1, 25%); intellectual disability (ID; n=1, 25%); essential tremor (ET; n=1, 25%); and a group of central nervous system disorders (CNSDs), including HD and PD (n=1, 25%).

**Figure 4:**
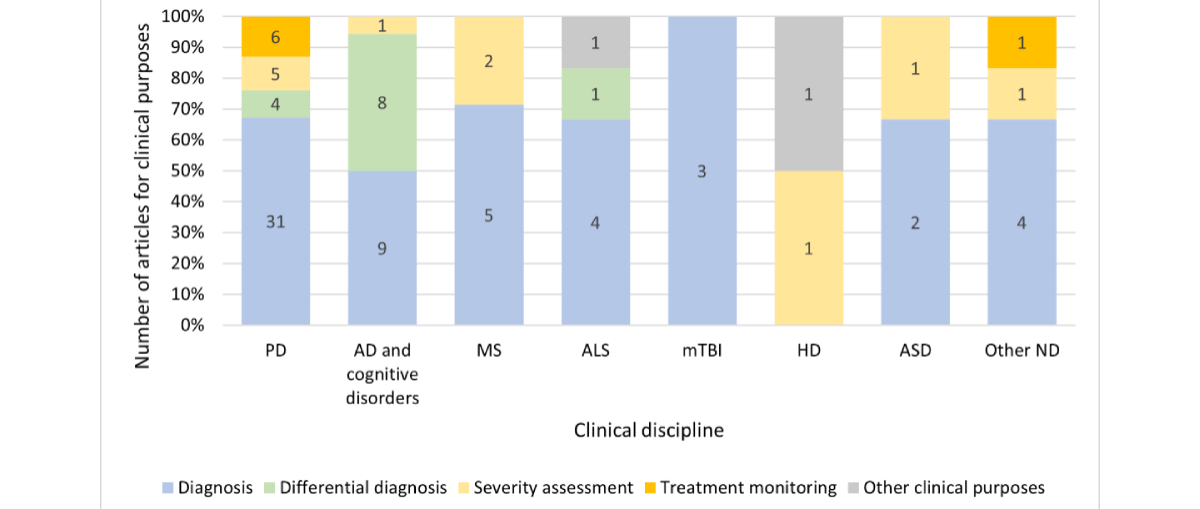
Distribution of the main clinical purposes based on disease categories. AD: Alzheimer disease; ALS: amyotrophic lateral sclerosis; ASD: autism spectrum disorder; HD: Huntington disease; MS: multiple sclerosis; mTBI: mild traumatic brain injury; ND: neurological disorder; PD: Parkinson disease.

Disease diagnosis, differential diagnosis, severity assessment, and treatment monitoring were the most mentioned clinical purposes in the studies. Figure 4 depicts the distribution of articles with their clinical purposes according to disease categories. Some studies (18/72, 25%) addressed multiple clinical purposes, such as diagnosis and differential diagnosis or diagnosis and severity assessment. Disease diagnosis was widely researched. These studies focused on prevailing clinical challenges such as the lack of definitive objective biomarkers, the need for noninvasive biomarkers, challenges in discriminating diseases with similar symptoms (differential diagnosis), and challenges faced by vulnerable populations such as older adults or rural populations.

#### PD Results

PD was the most investigated neurological condition in the studies (40/72, 56%). PD diagnosis, differential diagnosis, treatment monitoring, and severity assessment were explored. Many studies (28/40, 70%) investigated the diagnosis of PD by discriminating parkinsonian speech compared to healthy speech [42-69]. Several studies (3/40, 8%) carried out statistical comparisons of speech features in healthy and PD groups [70-72]. PD severity scores were predicted in the study by Viswanathan and Arjunan [73] for patients with PD and healthy controls. Early detection of PD was investigated in some studies (4/40, 10%) [42,47,57,65], in which participants with early PD were recruited to compare them with healthy participants. Early and mid-aged patients with PD were recruited in the studies by Montaña et al [65] and Wang et al [42], whereas the study by Lim et al [47] focused on patients with early- and advanced-stage PD according to the Hoehn and Yahr staging scale. Patients with PD were assessed in the study by Jeancolas et al [57] if they had been diagnosed with PD within 4 years before the study.

Some studies (4/40, 10%) investigated discrimination of similar-symptom diseases for differential diagnosis. For example, the study by Song et al [44] investigated distinguishing ataxic and hypokinetic dysarthria, which are commonly prevalent in neurodegenerative diseases. Patients with PD and cerebellar ataxia were considered representative cases of diagnosis. A statistical comparison of PD speech and MS speech was performed in the study by Vizza et al [72]. The challenge of differential diagnosis was addressed in the studies by Das et al [74] and Li et al [75] for atypical parkinsonian syndromes (APS) of progressive supranuclear palsy and multiple system atrophy.

In addition to diagnosis, the studies investigated PD severity predictions using the Unified Parkinson’s Disease Rating Scale (UPDRS) and Hoehn and Yahr scale. The studies by Viswanathan and Arjunan [73] and Hemmerling and Wojcik-Pedziwiatr [76] predicted UPDRS scores from the speech of patients with PD considering their medication status. Furthermore, Zhang et al [61] and Sztaho et al [67] predicted PD severity level from speech features in relation to the UPDRS and Hoehn and Yahr scale, respectively. The severity of PD symptoms was assessed in the study by Tunc et al [77] through collected speech samples at different points after taking levodopa.

The studies analyzed the speech of patients with PD to assess the impact of antiparkinsonian treatments on speech and voice function. For example, Suppa et al [43] studied speech changes during highly effective and less effective periods from levodopa therapy for PD. PD severity using UPDRS scores was predicted in the study by Hemmerling and Wojcik-Pedziwiatr [76] at different time points after taking levodopa. The effects of dopaminergic medication on speech function were investigated in the studies by Vandana et al [78] and Jain et al [79]. The impact of assistive speech devices in treating speech impairment was studied by Gaballah et al [80,81] considering these devices’ treatment capability outside the clinical facility. In these studies, Gaballah et al [81] investigated discrimination of PD speech and healthy speech under different environments and amplification conditions, whereas Gaballah et al [80] predicted the perceived voice quality of patients with PD with and without assistive speech amplifier devices.

#### AD and Cognitive Impairment Results

Cognitive decline was assessed through speech analysis, including clinical conditions such as AD, dementia, and MCI. Dementia presents different symptoms at different severity levels of cognitive decline. MCI represents an early stage of cognitive decline without interference with everyday life but can act as a transition stage between healthy aging and dementia when it acts as preclinical AD [116]. AD can evolve over a continuum from normal cognition to MCI due to AD, followed by more severe AD dementia [117]. AD is identified as the most common form of dementia [117].

The studies in this review analyzed speech at different cognitive decline stages for diagnosis, differential diagnosis, and severity assessment (12/72, 17%). The focus was on discriminating impaired cognition from normal cognition. The discrimination of patients with AD [82] and MCI [83-85] from healthy controls was explored. Nagumo et al [85] studied differential diagnosis with global cognitive impairment as they considered patients with MCI, global cognitive impairment, and both forms of cognitive decline. On the other hand, some studies (3/12, 25%) [86-88] investigated both diagnosis and differential diagnosis of AD compared to patients with MCI and healthy participants. Automatic differentiation of patients with MCI and early dementia from healthy controls was studied by Bertini et al [89].

Differential diagnosis of AD and dementia with Lewy bodies was investigated in the study by Yamada et al [90], whereas Sumali et al [91] worked in discriminating between patients with depression and dementia as certain mental disorders (eg, depression) can cause pseudodementia, a temporary decline in mental cognition. While Al-Hameed et al [92] discriminated speech from neurodegenerative diseases, including patients with AD, MCI, and dementia, from speech from functional memory disorder, König et al [93] predicted neuropsychiatric inventory scores in a sample of patients with MCI through speech.

#### MS Results

MS is a chronic inﬂammatory disease of the central nervous system that affects cognitive and motor functions causing motor and sensory impairments, visual disabilities, cognitive disorders, and speech and language deficits [118]. Different studies (5/72, 7%) investigated MS diagnosis and severity assessment through speech analysis. Discriminating patients with MS from healthy controls was investigated in several studies (4/5, 80%) [94-97], whereas Fazeli et al [94] explored the relationship between selected speech quality indexes and MS severity. Speech analysis among patients with MS at 2 different disease stages, in addition to comparisons of patients with MS versus healthy patients, was conducted in the study by Vizza et al [98].

#### ALS Results

ALS is a progressive motor neuron disease that affects upper and lower motor neurons in the motor cortex, the brain stem, and the spinal cord. ALS leads to muscular weakness and spasticity that can result in difficulties with mobility, breathing, and motor speech production [119]. Research on speech in ALS concentrated on identifying differential diagnoses and detecting bulbar involvement (4/72, 6%). Differentiation of speech from 3 participant groups, including patients with ALS with and without bulbar involvement and healthy controls, was conducted in the study by Tena et al [99]. The studies by Illa et al [100] and Likhachov et al [101] investigated discrimination of speech from patients with ALS and healthy participants. The ALS populations in both studies had shown signs of bulbar involvement. Furthermore, Mallela et al [102] investigated the diagnosis of ALS and differential diagnosis of ALS from PD by including ALS patients with a range of speech dysfunction severities.

#### mTBI Results

Compared to other progressive neurological diseases that occur due to a physical or mental phenomenon originating within the body, mTBIs or concussions are initiated when a person experiences an external force to the head, causing some alteration in brain function [120]. Therefore, the studies in this review on concussion (3/72, 4%) were able to access baseline speech recordings from highly vulnerable populations such as athletes along with the postinjury speech and then aimed to discriminate concussed speech from that of healthy controls [103] and from the individuals’ healthy baseline speech [104]. Concussion detection through baseline, postconcussion, and posthealthy speech comparison was also studied by Wall et al [105].

#### HD Results

HD is a rare severe neurodegenerative disease with a known natural history due to inheritance [106]. One study assessed the severity of HD, whereas the other study investigated the expression of emotions through vocal characteristics in participants with HD and pre-HD using emotion elicitation via speech. Riad et al [106] investigated the prediction of HD severity scores from speech features of a sample of participants with pre-HD and HD. Furthermore, they studied the relationship between speech variables and striatal volumes as well. In addition, the emotion expression capability of patients with HD was studied through speech-based emotion recognition [107].

#### ASD Results

ASD is a neurodevelopmental disorder that causes difficulties in social communication, particularly in verbal and nonverbal communication [121]. Most people living with ASD exhibit speech and expressive language abnormalities at different levels [121]. The 3% (2/72) of ASD articles in this review focused on discriminating ASD speech from healthy speech [108,109] and estimating autism severity by predicting Autism Diagnostic Observation Schedule scores from speech features [109]. It was noted that comorbid conditions were present in these populations, including attention-deficit/hyperactivity disorder [108] and children with suspicion of ASD, such as children with other language or developmental delays [109], in addition to children with typical development.

#### Other Neurological Disease Results

The neurological conditions that were the focus of other studies included ET (1/72, 1%), apathy (1/72, 1%), ID (1/72, 1%), and a broader category of CNSDs (1/72, 1%).

ET is among the most common tremor syndromes, which can encompass voice tremor as well [122]. To complement conventional neurological examination–based assessments, voice tremor in patients with ET was studied by Suppa et al [110] by discriminating the speech of patients with ET who did and did not manifest clinically overt voice tremor. Moreover, they discriminated between patients at baseline and after having medical treatments [110]. Apathy is identified as a motivation disorder that can present in several psychiatric and neurological conditions. Discrimination between patients with and without apathy was done in the study by König et al [111] through speech from patients with mild to moderate neurocognitive disorders. ID is a neurodevelopmental disease similar to ASD that causes cognitive delays in early childhood, resulting in delays in adaptive function, language, and speech [123]. Speech samples from children with typical development and those with ID were compared in the study by Aggarwal et al [112]. Lauraitis et al [113] investigated discrimination of speech impairment in patients with early-stage CNSDs from healthy speech. They included patients with HD, PD, cerebral palsy, stroke, and early dementia in their CNSD population.

### Speech Data

#### Overview

Across the 18.5% (72/389) of neurological studies, speech data were captured through a range of speech tasks, from simple production of sounds or words to spontaneous speech in conversations. The characteristics of the speech tasks impact the reliability of speech features [124]. For example, speech quality features from continuous speech may be less reliable compared to more structured sustained phonation tasks [124]. Therefore, speech tasks and speech features together define speech data in clinical speech analysis [14].

#### Speech Tasks

Speech tasks across the 18.5% (72/389) of neurological studies can be categorized along a continuum from highly constrained to naturalistic speech elicitation. At the most structured end are sustained phonations and diadochokinetic tasks. In the middle range are reading and picture description tasks, whereas prompted speech tasks elicit the most naturalistic speech production.

Highly constrained tasks frequently appeared in studies of neurological diseases. These included sustained phonation of vowels (/a/, /e/, /i/, /o/, and /u/) and sounds such as “ah,” “eh,” “iuh,” and “iamh.” Similarly structured were oral diadochokinetic tasks, including alternating motion rate and sequential motion rate tasks. Alternating motion rate tasks require rapid repetition of a single syllable (eg, /pa/, /ta/, or /ka/), whereas sequential motion rate tasks involve sequences of different syllables (eg, /pa-ta-ka/).

Semistructured tasks provided a balance between control and naturalistic speech production. Reading tasks typically included reading a set of words, sentences, and passages. Picture description tasks elicited spontaneous speech within a potentially limited vocabulary set associated with the provided visual aid. Activity-related speech included speech fluency tasks, recall and summary tasks, and number-related tasks such as counting and subtraction. On the other hand, prompted speech tasks encourage more free and spontaneous speech within a guided procedure. Monologues on own experiences, conversations including neurological examinations, and spontaneous speech induced by interactive questions were some examples. It was also common for the studies to use multiple speech tasks, combining structured and semistructured tasks. The studies used speech tasks from culturally adapted test batteries such as Thammasat-National Electronics and Computer Technology Center (NECTEC)-Chula’s Thai Language and Cognition Assessment [86].

Overall, structured speech tasks were more widely used for neurodegenerative diseases such as PD and ALS, whereas semistructured speech tasks were common for cognitive impairment–related disorders such as AD, MCI, and dementia. Textbox 2 shows the application of different types of speech tasks in the studies with examples.

**Types of speech tasks used in the included studies.**

**Structured tasks**

- Sustained phonation tasks: the participant is asked to produce a vowel, usually with a steady pitch, for several seconds.
  Sustained vowel: /a/ for multiple sclerosis (MS) [94,95], /a/ for amyotrophic lateral sclerosis (ALS) [101], /a/ for Parkinson disease (PD) [42,53,54,59,60,62,64,66,71,74,75,77,78], /e/ for PD [42,43,49], and /e/ for essential tremor (ET) [110]
  Group of vowels (/a/, /e/, /i/, /o/, and /u/): MS [98], ALS [99], and PD [46,55,61,72,76,79]
  Mix of phonemes and sounds: /s/, “sh,” and /f/ (ALS) [102]; continuous phonation task (intellectual disability [ID]) [112]; /a/, /o/, and /m/ (PD) [45]; /a/, /u/, and /m/ (PD) [70,73]; different sounds (“ah,” “eh,” “iuh,” and “iamh”) (PD) [51]; and /a/ and /u/ (PD) [56]
- Diadochokinetic tasks: the participant is asked to rapidly repeat alternating syllables (eg, /Pa/, /Ta/, and /Ka/) for several seconds.
  Monosyllables: /Pa/ and /Ka/ for mild traumatic brain injury (mTBI) [103]; /Pa/ and /Ta/ (PD) [55]; and /Pa/, /Ta/, and /Ka/ (PD) [52,63]
  Multisyllable sequence: /Pa-Ta-Ka/ for mTBI [103]; /Pa-Pa-Pa/, Ta-Ta-Ta/, Ka-Ka-Ka/, and Pa-Ta-Ka/ and /Ba-Da-Ga/ (ALS) [102]; /Pa-Ta-Ka/, /Pa-Ka-Ta/, /Pe-Ta-Ka/, and /Pe-Ka-Ta/ (PD) [79]; /Pa-Ta-Ka/ (PD) [65,74,75]; and /Pa-Ta-Ka/, /Pe-Ta-Ka/, and /Pa-Ka-Ta/ (PD) [52,63]

**Semistructured tasks**

- Reading tasks: the participant is provided with a predefined text to read out.
  Reading a set of words or sentences: set of words (MS) [94,97], set of words and sentences for mTBI [103], set of words and sentences for ALS [100], and reading sentences (PD) [42,43,46,49,50,52,54,55,57,60,63,66]
  Reading paragraphs: reading a Czech text (MS) [96], Sport Concussion Assessment Tool, Fifth Edition reading paragraph (mTBI) [105], read a short text from predeﬁned poems (central nervous system disorders) [113], read sentences from a French book (Alzheimer disease [AD]) [87], read a short passage (AD) [84], reading texts (PD) [44,52,55,57,58,63,74,79], and reading an article (PD) [47]
- Activity-based speech: The participant is asked to produce speech in response to a semistructured activity.
  Number-related tasks: digit words (mTBI) [104], counting forward and backward (Huntington disease [HD]) [106], counting [88,90] and subtraction for AD [90], and counting for PD [44,79]
  Recall and tell: summary of a story (MS) [97], immediate recall and delayed recall of a short film (AD) [83], and imitation of the instructor’s voice (ID) [112]
  Fluency tasks: verbal fluency task (AD) [88,90] and month-remembering task (PD) [79]
- Picture descriptions: the participant is asked to describe a visual aid presented to them.
  Picture description: cookie theft picture description [90] and picture description [86] for AD, and cookie theft picture description for PD [52,79]
- Prompted speech tasks: the participant is asked to produce a speech with the support of speech prompts.
  Monologue: talk about the previous day (MS) [97], a monologue (ALS) [100], monologue on a given topic (PD) [63,67,74,75], talk about positive and negative events in life (apathy) [111], talk about a positive event and negative event in life (AD) [93], and talk about the immediate day (AD) [83]
  Clinical interviews and conversations: interviews by neuropsychologists to describe the last 24 hours and tasks to elicit emotions (HD) [107], conversations during Autism Diagnostic Observation Schedule sessions (autism spectrum disorder) [108,109], conversations (PD) [80,81], and neuropsychological examinations and conversations (AD) [91,92]
  Spontaneous speech: speech induced through questions (eg, on a picture, about a working day, or about a dream; AD) [89] and 1-minute free talk with an artificial intelligence program (AD) [82]

#### Speech Features

The studies extracted speech features based on the characteristics of the speech tasks. From a signal processing perspective, the speech features included in the studies can be broadly categorized into 3 main types: fundamental and advanced signal processing–based speech features and audio images (Textbox 3). The primary difference between fundamental and advanced signal processing–based speech features lies in their capacity to correspond to phonetic aspects of speech production. This distinction is important for understanding how these speech features are explained from both a speech science and clinical perspective. Being single-dimensional, both these types of speech features are suitable candidates for statistical analyses, traditional ML approaches, and deep neural networks (DNNs). On the other hand, audio images, a form of multidimensional speech representation, act as candidates for image-specialized neural networks such as convolutional neural networks (CNNs).

**Speech feature types based on signal processing perspective.**

**Fundamental digital signal processing–based speech features**

- Mostly linear signal processing techniques
- Can be described based on signal representation dimension (eg, time domain or frequency domain)
- Can be mapped to a physiological phonetic viewpoint (eg, phonation or articulation) more straightforwardly
- Can be used to create secondary voice indexes, such as voice quality measures (eg, Dysphonia Severity Index)

**Advanced digital signal processing–based speech features**

- Mostly nonlinear signal processing techniques
- May not be directly mapped to a physiological phonetic viewpoint

**Audio images**

- 2D representation of speech signals

Speech features can be analyzed through 2 complementary lenses: their representation dimension during speech feature extraction and their physiological phonetic characteristics, which are derived from the field of speech science. Speech signals can be represented and features can be extracted in the time, frequency, time-frequency, and cepstral domains. From the physiological phonetic viewpoint, speech features capture biological and anatomical aspects of speech production, including articulation, phonation, prosody, and speech quality. Within the studies, speech articulation was assessed through time-domain features (eg, AMR [75], diadochokinetic rate, and diadochokinetic period [103]), frequency-domain features (eg, formants [42,76], Bark band energy-based features [42,54], spectral moment, and power spectral moment [76]) as well as cepstral domain features (eg, MFCC [42,45,54]). Similarly, speech phonation was assessed in features from time domain (eg, jitter, shimmer [42,45,62,73,76], amplitude and speed-based glottic cycle features [62,73], Teager-Kaiser energy operator based features [45,77], energy [76]), frequency domain (eg, fundamental frequency [42,48,62,73,76], bark band energy-based features [48], harmonic-to-noise ratio [45,73], noise-to-harmonic ratio [45]) and cepstral domain (eg, MFCC [48,53], linear-frequency cepstral coefficients [53], gamma-tone cepstral coefficients [53], cepstral peak performance [73]). Pause-based features [109] and fundamental frequency [109] are some examples for time domain and frequency domain features for prosody and rhythm assessment.

Different structures have been proposed for categorizing these features. Speech features were categorized as voicing, articulation, and prosodic features in the study by Li et al [75], whereas Wang et al [42] grouped speech features as phonatory, articulatory, prosodic, and cognitive-linguistic features. Phonatory features modeled abnormal patterns in the vocal fold vibrations, whereas articulation features captured deficits in articulatory movements of the lips, tongue, and jaw. Prosodic features, such as speech rate and timing, investigated paralinguistic aspects such as emotions, whereas cognitive-linguistic features considered vocabulary, phrase construction, and word repetitions [42]. The domain of speech feature representation pertains to the complexity and resource demands for feature extraction, whereas phonetic elements relate to speech task characteristics and the biological process involved in speech production.

Speech features are also categorized as linear or base speech features (eg, fundamental frequency, jitter, and shimmer) and nonlinear features, which are mostly derived from advanced signal processing techniques. The studies by Zhang et al [61] and Tunc et al [77] investigated nonlinear speech features such as correlation dimension, recurrence period density entropy, detrended fluctuation analysis, and pitch period entropy. Tunable Q-factor wavelet transform and empirical mode decomposition–based speech features are some more advanced speech features [77]. Different spectrograms, including mel spectrograms [44,54], linear spectrograms [54], and constant-Q transform spectrograms [54] are examples of speech image representations assessed in the included studies.

Secondary voice indexes were another type of speech feature available in the studies. They were indexes defined from other primary speech features. Example measures included the Dysphonia Severity Index, formant centralization ratio, and vowel metrics. The Dysphonia Severity Index is calculated from phonation time, jitter, fundamental frequency, and intensity, whereas the latter 2 are calculated from the measures of formants of vowels [72,94]. Some studies used standard speech feature sets, for example, the extended Geneva Minimalistic Acoustic Parameter Set [107], INTERSPEECH2016 Computational Paralinguistics Challenge speech feature set [49,110], and the emobase feature set [86]. Speech feature embeddings derived from a deep learning (DL) approach were also a type of speech feature representation assessed in the included studies [50,57].

### Data Science Approaches

#### Overview

Descriptive and predictive studies were included among the 18.5% (72/389) of neurological studies. Descriptive studies assessed the relationship between clinical variables and speech features (eg, whether speech features are associated with a disease or its severity), whereas predictive studies investigated the estimation of clinical variables from speech features (eg, predicting disease severity from speech features). Statistical analysis was largely applied in descriptive studies, whereas predictions were made through traditional ML and DL approaches.

#### Statistical Analysis: Relationship Between Speech Features and Clinical Measurements

Univariate, bivariate, and multivariate analyses of speech features were observed across the 18.5% (72/389) of neurological studies. Univariate analyses investigated descriptive measures and the statistical significance of speech features in differentiating pathological and healthy groups. Descriptive statistics of speech features within first hour and after 12 hours of medication of PD [78] and at 2 stages of MS [98] are some examples.

Further statistical significance tests showed the differences between speech features, mainly between healthy and impaired speech due to neurological health conditions. Several statistical significance tests were applied depending on the relationship between discriminating groups and the normality of the variables. For example, Suppa et al [110] statistically compared speech with and without voice tremors using the Student *t* test, whereas the paired Student *t* test was used to compare speech features before and after medication. To differentiate AD, dementia with Lewy bodies, and healthy speech, Yamada et al [90] compared speech features between groups after controlling for medication using 1-way analyses of covariance. Both studies explored the relationship between speech features and disease characteristics before building predictive models. The studies used bivariate analyses to explore the association between speech features and clinical variables, such as the correlation between speech measures and UPDRS scores for PD severity [73,76,77]. The study by König et al [111] also assessed the correlation between speech properties and the Apathy Inventory subscales to assess the properties of apathetic speech in people with cognitive disorders. Multivariate analysis was applied to simultaneously explore the relationship among clinical variables, speech features, and other confounding factors such as age and sex. For example, Svoboda et al [96] applied multiple linear regression of speech features, age, and sex to differentiate MS from healthy speech.

Table 1 summarizes the statistical analysis approaches applied in the selected studies.

**Table 1:** Summary of statistical analysis approaches in the studies with examples.

Technique			Examples
**Univariate analysis**			
	Statistical comparison of speech features between 2 independent groups	Mann-Whitney U test to compare speech features between patients with MS^a^ and healthy groups [94] Kruskal-Wallis test to compare apathetic and nonapathetic speech [111] Independent Student t test to compare speech features between patients with MS and healthy groups [95,98] and between patients with and without voice tremor [110] Kolmogorov-Smirnov 2-sample test to compare speech features in MS and healthy groups [96]	
	Statistical comparison of speech features between 2 related groups	Paired Student t test to compare the speech variables of the same patients with and without medication [110]	
	Statistical comparison of speech features among ≥3 independent groups	1-way ANOVA to explore how diseased status (AD^b^, DLB^c^, and healthy) impacted speech features to support differential diagnosis [90] 2-way ANOVA to examine the effects of the dementia type (AD and DLB) and disease stage (MCI^d^ and dementia) on speech features [90]	
**Bivariate analysis**			
	Statistical association between clinical variables and speech features	Spearman correlation coefficient to assess the relationship between acoustic features and neurological status for MS [94] and assess the relationship between acoustic features and Apathy Inventory subscales [111] Statistical association between ADOS^e^ score and speech features via Pearson correlation coefficient [109]	
**Multivariate analysis**			
	Statistical comparison among multiple groups considering confounders on speech features	Generalized linear regression to assess the statistical significance of speech features, age, and sex on differentiating MS against healthy populations [96]	
	Dimensionality reduction of speech features	Application of principal-component analysis and observation of biplots to understand speech feature clusters in 3 discriminative groups related to ALS^f^ [99]	
	Speech feature cluster analysis	k-means clustering to unveil patterns within speech features obtained from patients with dementia and depression [91]	

^a^MS: multiple sclerosis.

^b^AD: Alzheimer disease.

^c^DLB: dementia with Lewy bodies.

^d^MCI: mild cognitive impairment.

^e^ADOS: Autism Diagnostic Observation Schedule.

^f^ALS: amyotrophic lateral sclerosis.

#### ML Approaches

The studies used traditional ML, DL, and hybrid approaches to predict clinical variables from speech features. Disease diagnosis was formulated as a binary classification, whereas differential diagnosis was extended into multi-class classifications. The severity assessment was primarily treated as a regression problem, whereas treatment monitoring involved either regression or classification of patients based on medication status. Classification problems were the most commonly addressed.

Among traditional ML classifications, the following were the most common algorithms: support vector machine, k-nearest neighbor, random forest, decision tree, Extreme Gradient Boosting, multilayer perceptron, and logistic regression. Linear regression, support vector regression, random forest regression, and artificial neural network regression were among the traditional ML regression techniques, for example, in Autism Diagnostic Observation Schedule score prediction for autism severity assessment [109] and prediction of neuropsychiatric inventory scores [93].

DL approaches were applied as deep feature extractors and end-to-end predictors. In deep feature extractors, DL capability was explored in self-extracting efficient feature representations from speech features in a supervised or unsupervised manner. The studies retrained special DL models and built their own models as feature extractors. Example deep speech feature extractors included the implementation of a standard DNN in the study by Gosztolya et al [97] and an autoencoder network in the study by Bertini et al [89]. Regarding the development of end-to-end DL models, recurrent neural network models and CNNs were among the most promising ones. Different architectures such as CNN with a modified Hybrid Mask U-Net architecture with an adaptive custom loss function for PD assessment [46] and bidirectional long short-term memory neural networks [105] were implemented. Hybrid architectures such as CNN–long short-term memory networks [102] and personalized convolutional recurrent neural networks [79] were also assessed in the studies. Transfer learning was applied to train computer vision–based approaches for clinical speech analysis. Examples included CNN14 [44] and AlexNet-based CNN for PD assessment [53].

There were fewer research attempts to explore DL approaches for clinical score prediction. Autism severity score prediction using DNN and CNN [109] and PD severity prediction using DNN [67] were among the few examples. Table 2 summarizes the ML and DL approaches applied in the selected studies.

**Table 2:** Summary of machine learning (ML) and deep learning (DL) approaches used in the studies with examples.

Technique		Examples
**Traditional ML: classifiers**		
	Boosting technologies	XGBoost^a^ for MS^b^ diagnosis [96], AD^c^ assessment [82], and PD^d^ assessment [50,77] AdaBoost^e^ for AD and cognitive impairment assessment [86,92] and AdaBoost for PD assessment [47] LightGBM^f^ for PD assessment [47,50]
	KNN^g^	MS diagnosis [96], ALS^h^ assessment [101], AD assessment [87], and PD assessment [42,47,51,54,58,60,67,69]
	Simple feed-forward neural networks	MS diagnosis [96], PD assessment [54], and ID^i^ assessment [112] AD assessment [86], cognitive disorder assessment [92], and PD assessment [49,51,60,61,67]
	RF^j^	MS diagnosis [96], ALS assessment [99], and HD^k^ assessment [107] ID assessment [112], AD and cognitive disorder assessment [82,83,86,92], and PD assessment [47,50,66]
	SVM^l^	MS diagnosis [96], concussion diagnosis [104], ALS assessment [99,100,102], ASD^m^ assessment [108], ID assessment [112], ET^n^ detection [110], AD and cognitive impairment assessment [83,86-88,90-92], and PD assessment [43,45,47,49-51,53,54,58-61,63,67-69,74,75]
	LR^o^	Concussion diagnosis [103], diagnosis of apathy [111], ALS assessment [99], AD assessment [82,86], and PD assessment [42,47,59,61,75]
	NB^p^	ALS assessment [99], AD and cognitive disorder assessment [83], and PD assessment [42,47,49,54,60,61]
	DT^q^	AD assessment [87] and PD assessment [47,54]
**Traditional ML: regressors**		
	Linear regression	ASD severity prediction using multiple linear regression [109] NPI^r^ score prediction using linear regression with L1 regularization [93] UPDRS^s^ score prediction for PD assessment [61,76] HY^t^ score prediction for PD assessment [67]
	SVR^u^	ASD severity prediction [109] NPI score prediction [93] UPDRS score prediction for PD assessment [61,73,76] HY score prediction for PD assessment [67]
	RF regression	UPDRS score prediction for PD assessment [73,76]
	ANNs^v^	Prediction of speech impairment in ET [110] Prediction of the likelihood of speech impairment in PD [43] HY score prediction for PD assessment [67]
**DL and hybrid models: classifiers**		
	DNNs^w^	DNN+SVM model for MS diagnosis [97] ALS assessment [100,102] AD and cognitive impairment assessment [84] X-vector for PD assessment [57] DNN for PD assessment [50,67]
	RNNs^x^	BiLSTM-A^y^ for concussion diagnosis [105] BiLSTM^z^ for CNSD^aa^ diagnosis [113] and PD assessment [54]
	CNNs^ab^	AlexNet-based CNN for PD assessment [53] CNN14 for PD assessment [44] CNN (modiﬁed Hybrid Mask U-Net architecture with an adaptive custom loss function) for PD assessment [46] CNN (ResNet18 architecture) for PD assessment [52] CNN for PD assessment [54,58,63]
	CNN+LSTM^ac^	CNN+LSTM model for ALS assessment [102] CRNN^ad^ for PD assessment [79]
	Autoencoder	auDeep+MLP^ae^ network for early dementia assessment [89]
**DL and hybrid models: regressors**		
	DNNs	ASD severity prediction [109] DNN regression [80] HY score prediction for PD assessment [67]
	CNNs	ASD severity prediction [109]

^a^XGBoost: Extreme Gradient Boosting.

^b^MS: multiple sclerosis.

^c^AD: Alzheimer disease.

^d^PD: Parkinson disease.

^e^AdaBoost: Adaptive Boosting.

^f^LightGBM: Light Gradient-Boosting Machine.

^g^KNN: k-nearest neighbor.

^h^ALS: amyotrophic lateral sclerosis.

^i^ID: intellectual disability.

^j^RF: random forest.

^k^HD: Huntington disease.

^l^SVM: support vector machine.

^m^ASD: autism spectrum disorder.

^n^ET: essential tremor.

^o^LR: logistic regression.

^p^NB: naive Bayes.

^q^DT: decision tree.

^r^NPI: neuropsychiatric inventory.

^s^UPDRS: Unified Parkinson’s Disease Rating Scale.

^t^HY: Hoehn and Yahr scale.

^u^SVR: support vector regression.

^v^ANN: artificial neural network.

^w^DNN: deep neural network.

^x^RNN: recurrent neural network.

^y^BiLSTM-A: bidirectional long short-term memory–attention.

^z^BiLSTM: bidirectional long short-term memory.

^aa^CNSD: central nervous system disorder.

^ab^CNN: convolutional neural network.

^ac^LSTM: long short-term memory.

^ad^CRNN: convolutional recurrent neural network.

^ae^MLP: multilayer perceptron.

Some studies (24/72, 33%) integrated multiple data science approaches. They first applied statistical techniques to screen and filter speech features before feeding them into ML-based prediction models. Application of unsupervised learning techniques was the least common, with only one study exploring unsupervised clustering. The study applied statistical analysis, unsupervised learning, and supervised classification to address the classification of patients with depression and dementia based on speech, demonstrating comprehensive use of data science approaches in the speech analysis pipeline [91].

### Model Evaluations

The analytical performance of algorithms was evaluated based on the model’s prediction performance, generalizability, robustness, biases, and fairness. The classification model’s prediction performance was assessed through a set of performance metrics, including accuracy, sensitivity, specificity, and the area under the receiver operating characteristic curve. In regression analysis, the root mean square error score was the most widely reported performance metric.

To ensure the model’s generalization and robustness, cross-validation with speaker independence was applied to prevent overfitting. Variations of k-fold validations were applied, with *k*=5 and *k*=10 being the most common. In training and testing, speaker independence maintains all speech recordings of the same speaker only in either the training or testing set without splitting between the 2 to avoid overoptimistic performance from information sharing. The studies adapted speaker-independent cross-validation strategies with simple k-fold validation when utterance-level features were considered. Some studies (14/72, 19%) applied leave-one-out cross-validation to increase the training dataset, preserving speaker independence. In this case, data from one participant were kept for testing, whereas all remaining data were used for model training [45,67,83,93]. When low-level speech features such as frame-level speech features were analyzed, speech from the same utterance was treated as a separate input, making speaker independence challenging. To address that challenge, Al-Hameed et al [92] applied a leave-one-group-out cross-validation with segment-level speech features, whereas simple k-fold validation was used with utterance-level features.

The biases and fairness of model predictions were mainly evaluated through cross-corpus testing and confounder assessments. There were several cross-corpus test scenarios in the studies. Training with aged-matched groups and testing on different age groups [91] and recruiting different training and testing cohorts [47] were some examples of maintaining different corpora within data collection. Moreover, the studies combined public and private datasets from different ethnicities and different speech tasks to improve the heterogeneity of the sample populations [46,58,61,63,77]. Speech corpora with different speech recording qualities were also considered [48,52,55,57,59].

The impact of confounding factors such as age and gender were addressed differently in the studies. Mainly, the studies used age-matched disease and control groups in the experiments. Meanwhile, age mismatch between diseased and healthy groups was addressed through age correction of speech features [99]. Application of age and gender as features in their models [84,96] as well as evaluation of model performance for each gender was also conducted [111].

**Figure 5:**
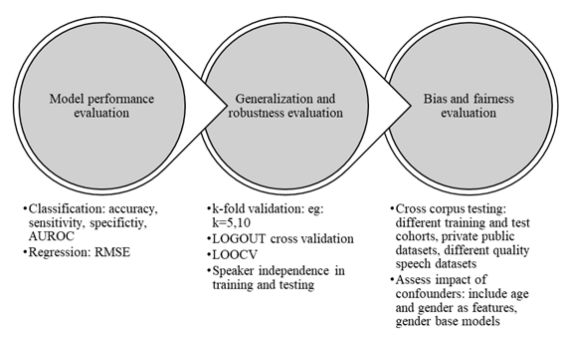
Model evaluation criteria used in the studies on neurological populations. AUROC: area under the receiver operating characteristic curve; LOGOUT: leave-one-group-out cross-validation; LOOCV: leave-one-out cross-validation; RMSE: root mean square error.

Figure 5 shows the model evaluation criteria commonly considered in the studies.

Only one study evaluated model predictions within a clinical setting. To discriminate between ataxia and hypokinetic dysarthria, Song et al [44] compared predictions of their artificial intelligence models and clinical decisions from a group of neurological resident physicians.

### Clinical Applications

Several studies (6/72, 8%) extended their research into applications in the PD, ALS, and mTBI domains. For example, Rahman et al [50] proposed a web-based framework to record and analyze speech for PD screening. Meanwhile, Zhang et al [61] deployed their real-time speech analysis tool for PD diagnosis and severity assessment within a mobile app called No Pa for both Android and iOS. In addition, Likhachov et al [101] created a prototype of a mobile app named ALS Expert to assess the voice function of ALS. The studies used mobile apps for their speech data collection to demonstrate the feasibility of remote assessments and remote monitoring. For example, Vasquez-Correa et al [52] collected smartphone-based speech data via an app called Apkinson, and Laganas et al [48] used a mobile app named iPrognosis to collect speech data from several countries and adopted an on-device feature extraction process. Both studies focused on PD assessment. A lightweight mobile app for speech data collection for mTBI assessment was also developed in the study by Daudet et al [103] to demonstrate the feasibility of on-device speech feature extraction, highlighting the importance of speech analysis at resource constraint devices to support on-field assessments.

## Discussion

### Principal Findings

On the basis of our review, speech analysis has emerged as a valuable tool across neurological, psychiatric, and respiratory diseases, with a particular focus on PD, AD, and cognitive impairment. Research in speech analysis has spanned the continuum of patient care, addressing diagnosis, differential diagnosis, severity assessment, and treatment monitoring. However, much research was conducted on diagnosing diseases by classifying healthy and diseased populations. Prediction of continuous clinical variables such as clinical scores was explored through regression. Statistical analysis was applied to assess the reliability of speech features and their relationship with clinical variables.

However, there are several limitations in the current research approaches to make their transition into health care settings. Conducting in controlled settings with homogeneous populations, typically single-ethnicity, single-center cohorts was observed as a common limitation across the studies. Early-stage patients—often the most challenging to diagnose—were underrepresented, and longitudinal studies across disease progression were scarce. However, the presence of studies from countries such as Italy, Thailand, China, Spain, and India with speech analysis in local languages is encouraging. This is appealing as speech features may or may not have similar meanings across different languages due to phonetic differences. Therefore, speech assessments within different populations and languages are recommended to assess the generalizability of speech analysis to a wider population [46]. Embedding heterogeneity into study populations and speech recordings through cross-corpus testing, using multiple speech tasks, and using different speech recording conditions were presented in the studies.

The diversity in the speech tasks used and extraction of speech features reflects both the field’s complexity and its opportunities. The studies used multiple speech tasks as separate modalities, ranging from highly structured exercises to naturalistic speech. The speech tasks within the studies could largely be categorized into reading tasks, sustained phonations, diadochokinetic tasks, activity-related speech tasks, picture descriptions, and prompted speech tasks. The selection of speech tasks was guided by disease characteristics as structured speech tasks were common for neurodegenerative diseases such as PD and ALS, whereas semistructured speech tasks were common for cognitive impairment–related disorders such as AD, MCI, and dementia. Sustained vowel phonation and diadochokinetic tasks were widely identified in the literature for their ability to represent early signs of neurological diseases [125] and generally demand lower cognitive demands than semistructured speech tasks such as reading tasks. Sustained vowels can be applied across different languages as well due to less linguistic loading related to dialect, region, and language [126]. However, prompted speech tasks such as monologues might be impacted by personality traits, emotional status, sociocultural norms, and the ability to tell stories [124]. In speech feature extraction, the studies reported various associated parameters such as speech frame sizes, window overlapping, audio preprocessing stages, and signal processing algorithms, but no common format exists to present speech feature characteristics to act as biomarkers. When positioning speech features as a biomarker, it is recommended to report parameters associated with speech-processing techniques as they affect the accuracy and robustness of acoustic measures [124]. Adhering to the established guidelines for speech recording and analysis [127,128] along with transparent reporting of the relevant instrumental and computational specifications can lead to reliable data collection and analysis. This facilitates comparisons across studies, accelerates knowledge sharing across interdisciplinary fields, and supports research advancement and reproducibility.

Among data science approaches, statistical analysis approaches were applied to assess the clinical utility of speech features, but the exploration of unsupervised approaches was less present. Traditional ML approaches dominated predictive studies, but there are growing efforts in applying DL approaches with complex network architectures, transfer learning approaches, and audio image analysis. Although transfer learning from computer vision to audio images was explored, there is a void in transferring knowledge between speech analysis in different languages. Despite their limited presence, DL implementations encompassed end-to-end implementations, deep acoustic feature extraction, and transfer learning, which represent the main stems of DL approaches [129]. Among the end-to-end implementations, CNN, long short-term memory, and hybrid implementations were used, but it was noted that no transformer-based neural network models were included in the selected studies. However, recent literature provides evidence of the application of vision transformer–based models to analyze speech signals for neurological diseases. Differentiating PD severity levels using sustained vowels and Swin Transformer [130]; differentiating speech from neurological diseases, including PD and MS, from healthy speech by retraining Google’s Vision Transformer Base model [131]; and differentiating PD speech from spinocerebellar degeneration speech by retraining the Patchout faSt Spectrogram Transformer [132] are some examples of vision-based transformers in speech analysis. It is noteworthy to mention that exploration of advanced DL approaches may improve the prediction performances. Additionally, incorporating a range of data science methods such as statistical analysis and unsupervised learning for hypothesis development and testing, conducting confounding factor analysis, and developing interpretable ML models can help researchers present their findings more interpretably and comprehensively to a wider audience.

Model evaluations in the included studies mainly considered analytical validations on prediction performance, generalization and robustness, and biases and fairness. Lack of clinical validations and narrow model assessment scopes can hinder translating research outcomes into clinical practices in the foreseeable future. Only a few studies (6/72, 8%) connected their investigations to clinical supportive applications. However, the development of applications can support further studies and usability assessments of prospective services within clinical and natural environments. In clinical settings, speech assessments can be conducted following standard speech recording protocols in optimal acoustic conditions to convey clinical insights for decision support. Routine health examinations might also be integrated with speech-based assessments to provide cost-effective longitudinal evidence in patient monitoring. Implementation of smartphone apps for speech collection, speech analysis, or transfer of speech data to remote clouds enables speech analysis in natural environments to empower telemedicine platforms. Opportunities exist in using telemedicine in neurological health care to ease the financial and accessibility burdens, such as in the rehabilitation of brain injuries and on-site concussion assessments [133]. Extending research into testable applications helps researchers explore challenges when converting research outcomes into clinical practice. Therefore, we believe that implementing a systematic research process leading to a clinical utility analysis could significantly accelerate advancements in this field. To support this, we organized the key concepts of speech analysis identified in this review into a research framework, aiming to provide more comprehensive guidance for future research.

### Proposed Research Framework

#### Overview

We adapted the research process proposed in the study by Offermann et al [134] to operationalize research in design science. The proposed research process is structured in 3 main phases—problem identification, solution design, and evaluation—supporting both quantitative and qualitative research methods [134]. To build our research framework, we identified key activities and desired outcomes at each phase, focusing on research using primary speech data collection. Table 3 shows the research process, including proposed activities under each subprocess of the main stages of problem identification, solution design, and evaluation. Expected outcomes are proposed at each stage to guide the research progress.

**Table 3:** Proposed research framework for speech analysis for clinical assessments.

Subprocesses and activities	Outcome
**Problem identification**	
Literature research and expert interviews Identify clinical challenges of interesting health conditions Characterize the speech impairment associated with the health condition through evidence from clinical science, speech science, and empirical research Identify the problem Identify a research gap Pre-evaluate relevance Define the research objective Define research questions Develop a research hypothesis	Selected health conditions and clinical purposes Potential clinical applications Characteristics of speech impairment Research hypothesis
**Solution design**	
Literature research Identify standard clinical assessments Identify speech assessments associated with specific health condition or similar health conditions Identify study design approaches and research techniques Identify suitable public or accessible datasets Design artifacts Define ML^a^ problem for the focused clinical purpose Develop the study design of the research Carry out data collection and analysis	Study population Participant recruitment and selection Data collection protocol Ground truth Demographic data Speech tasks and speech features Data analysis Primary analysis to build speech feature dataset Secondary analysis to assess the relationship between speech features and clinical variables Tertiary analysis to build predictive models
**Evaluations**	
Refine hypothesis Refine hypothesis based on data analysis Laboratory experiments Internal validation (evaluate predictive performance; extend evaluations beyond predictive performance [eg, evaluate biases, fairness, and explainability]) External validation (with public dataset if available) Case study and expert survey Case study in a clinical environment Qualitative feedback from clinical and speech science experts	Internal validation of results External validation of results Case study and clinical feedback Limitations and further improvements

^a^ML: machine learning.

#### Problem Identification

Problem identification defines the diseases of interest, clinical purpose, and potential clinical applications. Furthermore, it helps characterize the speech impairment associated with the disease to formulate research hypotheses.

As per this review’s findings, digital clinical speech analysis addresses different clinical problems. For example, speech features are being researched as objective biomarkers for disorders such as AD, mTBI, and PD in cases in which a definite biomarker is not available. Moreover, speech analysis aims to empower the remote monitoring of patients with PD who are mostly older adults. Speech analysis tries to differentiate overlapping symptoms in different diseases, such as cognitive impairments and symptoms of natural aging. Therefore, researchers can focus on a particular health condition considering existing clinical challenges and evidence of associated speech impairments [114]. To explore clinical challenges and potential speech changes from diseases, existing literature and experiences from health care experts such as clinicians, speech-language pathologists, and frontline health professionals can be referred. Experiences from patients might also be beneficial when the target end applications are considered. Findings of this phase identify a research gap and define research objectives and research questions. Furthermore, the researcher can qualitatively characterize speech impairments and define research hypotheses for empirical research.

#### Solution Design

In the solution design phase, specific literature research on current clinical practices and associated clinical speech assessments is beneficial to identify the ground truth [114] and potential speech characteristics to focus on. Study design approaches and state-of-the-art data analysis techniques can be used to ensure the scientific validity and novelty of the research. Clinical problems can be mapped into a predictive modeling problem, and an appropriate speech data collection plan can be developed. Data collection should include patients’ demographics, assessment of health conditions through standard clinical measures, and speech recordings. Furthermore, it should define speech recording instances, conditions, recording equipment, and speech tasks. Recording instances define when to collect speech from the participants, such as before or after medication. Recording can be done in either controlled or uncontrolled environments based on the research objectives. For example, if the research objective is the remote monitoring of patients, recordings in an uncontrolled environment would be more appropriate. Speech tasks should be carefully selected to capture appropriate speech characteristics associated with the health condition.

We recommend a comprehensive analysis of speech features to conduct association analysis to clinical parameter predictions. The data analysis workflow can be divided into three main stages—primary analysis, secondary analysis, and tertiary analysis—to (1) explore participant characteristics and extract suitable speech features from speech signals, (2) examine the relationship between speech features and clinical variables, and (3) develop and optimize ML models to predict clinical variables from speech features.

The primary analysis quantifies speech features from the speech signals following appropriate audio preprocessing. Noise reduction, down sampling, and silent period removals are some examples of audio preprocessing steps. Different speech features represent different aspects of speech production. For example, energy-based speech features represent respiratory function, whereas pitch-based features represent phonatory function. On the basis of the anticipated speech impairment and speech task characteristics, a set of representative speech features could be extracted. The secondary analysis then encompasses the relationship between speech features and clinical variables through descriptive statistics, statistical comparisons, association mining, and unsupervised data explorations.

In tertiary analysis, research can exploit speech variability within diseases to build predictive models, typically through classification or regression. Insights derived from secondary analysis can be integrated into predictive model building through feature selection and patient clustering. In addition, the impact of confounding factors such as age and gender should be considered when developing predictive models. Algorithm-dependent advanced ML strategies such as feature selection, data augmentation, and transfer learning can be explored to improve model predictions.

#### Evaluation

Once the solution has reached a satisfactory state, the evaluation of the proposed solutions or approaches is recommended [134]. During evaluations, the hypothesis can be refined to a more precise level based on the data analysis. For example, data analysis might highlight speech impairments in a particular dimension of speech production, such as phonation or articulation, or a particular stage of disease. Therefore, specific hypotheses can lead to detailed insights. Moving forward, internal validations should extend to different dimensions of model performance, and evaluations can include surveys and case studies for clinical utility assessments.

Through a set of comprehensive experiments and evaluation metrics, internal validations should extend beyond prediction performance to include assessments of biases and fairness, reliability, and explainability of predictions to address challenges that persist in speech analysis [28]. With the aid of publicly available datasets or, if feasible, with external cohorts, external validation of predictions can be conducted. Few case studies can be carried out in a clinical environment to present model assessments to clinical experts and obtain their feedback on model usability.

Finally, study results can be presented including experimental results for internal validations, external validations, and feedback from expert observations. We believe that the research outputs will be more competent and thorough and contribute to long-term research directions, extending the short-term results of the specific research scope.

### Limitations

We acknowledge that our research study has certain limitations. Among the eligible studies, we reviewed only 72 articles in the neurological domain. Although we considered only content-independent speech analysis, content analysis can also be relevant for certain clinical conditions. Furthermore, our main findings were synthesized from studies that conducted primary speech data collection. Nonetheless, studies on other clinical disciplines and studies that used publicly available datasets also contribute to the advances in the field. We confined the main concepts to diseases, clinical outcomes, speech tasks, speech features, data science approaches, evaluations, and clinical applications. The highly technical and algorithm-dependent speech feature extraction and feature selection methods were not covered in this review. However, such factors also remain crucial in speech analysis.

### Conclusions

This review discussed the main concepts within the growing research field of speech analysis for clinical decision support. The principal findings were presented from both clinical and technical perspectives. The clinical context was addressed through diseases, clinical purposes, and clinical applications, whereas technical aspects were addressed through speech tasks, speech features, data science approaches, and model evaluations.

The main contribution of this research can be summarized as (1) carrying out a comprehensive and extensive systematic scoping literature review followed by qualitative content analysis on digital clinical speech analysis and (2) presenting a research framework on speech analysis for clinical decision support.

The findings of this research reflect the potential of speech analysis for clinical decision-making and the contribution of data science approaches. Among clinical disciplines, neurological diseases have gained major interest, with PD being the most popular. Interestingly, research efforts are expanding beyond English-speaking populations, but more studies including less represented ethnicities and languages are much warranted. The lack of longitudinal studies also remains as a research gap. Designing experiments to address challenging clinical decision scenarios such as prognosis or early detection might be more appealing for clinical environments. Moreover, given the technical differences in speech features, an interpretable presentation of speech features as a digital biomarker would accelerate research progression and reproducibility. Integration of different data science techniques, including statistical analysis and unsupervised and supervised learning, can make data analysis more comprehensive and interpretable. Model evaluations should expand beyond analytical validations and include more comprehensive evaluations, including clinical utility assessments.

On the basis of the findings of this study, we proposed a research framework for primary research on speech analysis for clinical decision support. We encourage studies to adhere to design science research methodology by integrating both quantitative and qualitative research methods.

## Data Availability

All data generated or analyzed during this study are included in this published article and Multimedia Appendix 3.

## Appendix

Multimedia Appendix 1 PRISMA-ScR (Preferred Reporting Items for Systematic Reviews and Meta-Analyses extension for Scoping Reviews) checklist.

Multimedia Appendix 2 Search strategy.

Multimedia Appendix 3 Overview of the studies included in this review.

## Abbreviations

AD: Alzheimer disease
ALS: amyotrophic lateral sclerosis
ASD: autism spectrum disorder
CNN: convolutional neural network
CNSD: central nervous system disorder
DL: deep learning
DNN: deep neural network
ET: essential tremor
HD: Huntington disease
ID: intellectual disability
MCI: mild cognitive impairment
ML: machine learning
MS: multiple sclerosis
mTBI: mild traumatic brain injury
NECTEC: National Electronics and Computer Technology Center
PD: Parkinson disease
PRISMA-ScR: Preferred Reporting Items for Systematic Reviews and Meta-Analyses extension for Scoping Reviews
UPDRS: Unified Parkinson’s Disease Rating Scale
