# Supplementary material for: Clinical Decision Support Using Speech Signal Analysis: Systematic Scoping Review of Neurological Disorders
Source: J Med Internet Res. 2025 Jan 13;27:e63004. doi: 10.2196/63004 (PMC11773292; doi:10.2196/63004)
Supplement: Multimedia Appendix 2 [file jmir_v27i1e63004_app2.docx]

**Multimedia Appendix 2: Search strategy for databases**

The set of keywords under four main concepts were grouped in different ways to form search queries. Those search queries were tested on selected academic databases/search engines, to come up with the optimized search query for each platform. Three main formats of search queries were applied. Table II documents applied search query and return record count from each information source within stipulated limits/filters.

**Search Query 1:** (audio OR voice OR acoustic OR speech OR sound OR vocal ) AND ( analy* OR process* OR assess* OR recogni* ) AND ( health* OR medical OR clinical ) AND ("artificial intelligence" OR "machine learning" OR "deep learning" OR "statistical analysis" OR "data science")

**Search Query 1_GS:** (audio OR voice OR speech OR vocal ) AND (analysis OR analytics OR analytic OR process OR assess) AND ( health OR medical OR clinical ) AND (“artificial intelligence” OR “machine learning” OR “deep learning”)

**Search Query 1_SD:** (audio OR voice OR speech OR vocal ) AND ( analysis OR analytics OR process OR assess ) AND ( health )

Table S1 depicts the search query used for each database.

| # | Data Source/Search Engine | Search Query | Limits and Filters |
| --- | --- | --- | --- |
| 1 | Scopus | **Search Query 1:** | Fields: Title, Abstract, Keywords Filters: Year and Language |
| 2 | Google Scholar | **Search Query 1_GS** | Review articles.  Filters: Year |
| 3 | Medline via PubMed | **Search Query 1:** | Fields: Title/Abstract, Text word  Filters: Year |
| 4 | SpringerLink | **Search Query 1:** | Fields: Any  Filters: Year, Language, Articles and Conference papers |
| 5 | ScienceDirect | **Search Query 1_SD** | Filed: Title, Abstract, Author-specified keywords  Filters: Year |
| 6 | IEEE Xplore | **Search Query 1:** | Fields: Any Filters: Year, Conferences, and journals |
