# Supplementary material for: Clinical Decision Support Using Speech Signal Analysis: Systematic Scoping Review of Neurological Disorders
Source: J Med Internet Res. 2025 Jan 13;27:e63004. doi: 10.2196/63004 (PMC11773292; doi:10.2196/63004)
Supplement: Multimedia Appendix 3 [file jmir_v27i1e63004_app3.docx]

# **Multimedia Appendix 3: Table S1**

Summary of included studies in this review (n=72)

## **Parkinson’s Disease (PD)**

|  | **Study** | **Year** | **Disease** | **Clinical purpose** | **Country/Language** | **Sample size** | **Speech test** | **Speech features** | **Ground truth** | **Data science approach** |
| --- | --- | --- | --- | --- | --- | --- | --- | --- | --- | --- |
| 1 | Wang et al. 2022 [44] | 2022 | PD | - Early detection | China | - Single-ethnicity-single centre - 50 are diagnosed with PD - 50 healthy people | - sustain vowel: (“aaa. . .” and “eee. . .” in Chinese Pinyin), - two short sentences | - Phonation-based features: Jitter, Shimmer, Pitch Perturbation Quotient (PPQ), Amplitude Perturbation Quotient (APQ), Fundamental Frequency based features, Logarithmic Energy (LogE). - Articulation features: Bark Band Energies in onset and offset transitions, MFCC-based features on the onset and offset transitions, F1 and F2-based features. | - Healthy - Early and mid-PD (HY staging scale 1–1.5 as early stage and 2– 3 as the middle stage) | **Classification**   - Naïve Bayes (NB) - K-Nearest Neighbor (KNN) - Logistic Regression (LR) - Stochastic Gradient Descent (SGD)   10-fold cross-validation (CV) |
| 2 | Suppa et al. 2022 [45] | 2022 | PD | - Diagnosis - Treatment monitoring | Italy | - Single ethnicity - 115 PD patients - Patients: 57-Early stage, 58-mid stage - 31 mid-stage patients in ON and OFF therapy - 108 healthy subjects | - sustained vowel /e/ - Italian sentence | - INTERSPEECH2016 Computational Paralinguistics Challenge (IS ComParE 2016) feature dataset | - Healthy - Early-stage PD (HY ≤2), - mid-advanced-stage PD (HY >2) - OFF therapy: after at least 12 h of medication - ON therapy: 1–2 h after medication | **Classification**   - Support Vector Machine (SVM)   **Estimate voice impairment likelihood.**   - Artificial Neural Networks (ANN)   10-fold CV |
| 3 | Song et al. 2022 [46] | 2022 | PD,  cerebellar ataxia | - Diagnosis - Differential Diagnosis of dysarthria | - Korea | - Single-centre - Paragraph reading: - 76: no dysarthria speech - 112: ataxia speech - 207: hypokinetic dysarthria speech. - Number reading: - 82: no dysarthria speech - 111: ataxic speech - 216: hypokinetic dysarthria speech | - numbering from 1 to 50 - A paragraph with 9 sentences about autumn | - Log-Mel spectrograms | - Healthy - Ataxia dysarthria - Hypokinetic dysarthria. | **Classification**   - Convolution Neural Networks (CNN14)   5-fold CV |
| 4 | Motin et al. 2022 [47] | 2022 | PD | - Diagnosis | Australia | - Single center - 36 PD patients - 36 healthy (aged matched) | - 3 sustained phonemes: /a/, /o/, and /m/ | - Jitter parameters. - Shimmer parameters. - Teager-Kaiser energy operator (TKEO) - HNR and NHR - Glottal Quotient (GQ) - Glottal to Noise Excitation ratio (GNE) - Vocal fold excitation ratio (VFER) - MFCCs | - Healthy - PD | **Statistical analysis**   - Mann-Whitney U test for group difference and feature significance   **Classification**   - Support Vector Machine (SVM)   leave-one-out validation |
| 5 | Maskeliūnas et al. 2022 [48] | 2022 | PD | - Diagnosis | - Lithuania - Italian public corpus | - Single centre, multiple ethnicity with public data - Lithuanian - 61 PD (28 males and 33 females) - 43 healthy - Italian - 28 PD (19 men and 9 women) - 22 (10 men and 12 women) healthy | - phonetically balanced Lithuanian sentence (Lithuanian) - ﬁve sustained vowels (Italian) | - MFCC pictures | - Healthy - PD (physically independent patients up to stage 3 on HY) | **Classification**   - Convolution Neural Network - modiﬁed Hybrid Mask U-Net architecture with an adaptive custom loss function |
| 6 | Lim et al. 2022 [49] | 2022 | PD | - Diagnosis/Early Diagnosis/Pre-Diagnosis | - Taiwan | - Multiple cohorts, single center   Training cohort   - 112 PD during ‘on; phase (Early stage=70, advanced stage=42) - 111 healthy   Validation cohort   - 74 PD patients during the “off” phase or drug naïve (early stage=49, advanced stage=25) - 74 controls | - read an article | - reading time - phonetic score - pause percentage. - voice volume variance. - average pitch - pitch variance | - Healthy - Early-stage PD: HY <3 - Advanced-staged PD: HY ≥3. - “on” phase: within 3 hours of dopaminergic medication - “off” phase: more than 12 h after the last dose of dopaminergic medication | **Statistical analysis**   - Variable comparison with two-tailed t-tests or analysis of variance (ANOVA) or non-parametric t-test   **Classification**   - C4.5 Decision Tree - k-Nearest Neighbour (KNN) - Support Vector Machine (SVM) - Naïve Bayes (NB) - Random Forest (RF) - Logistic Regression (LR) - Gradient Boosting Machine Classiﬁer - AdaBoost - Light Gradient Boosting Machine   10-fold CV |
| 7 | Laganas et al. 2022 [50] | 2022 | PD | - Diagnosis | - English/Greek/German/Portuguese across multiple countries | - Multiple locations - Training: crowd-source data collection - 106 PD - 92 healthy - English speakers (54 PD, 36 HC) - German speakers (24 PD, 93 HC) - Greek speakers (15 PD, 150 HC) - Portuguese speakers (13 PD, 113 HC) - Testing dataset - 39 PD - 24 healthy - English speakers (10 PD, 10 HC) - Greek speakers (14 PD, 6 HC) - German speakers (15 PD, 6 HC) | - 15-75 s of running speech during phone calls via the iPrognosis app | - Fundamental frequency - MFCCs - Bark-band Energies (BBE) | - Healthy - PD - Language | **Classification**   - Single Instance learning (SIL) - Linear Support Vector Machine (LSVM) - Logistic Regression (LR) - Random Forest (RF) - Multiple Instance learning (MIL) - Normalized Set Kernel (NSK) - Statistic Kernel (STK) - sparse MIL (sMIL) - multiple instance SVM (mi-SVM)   leave-one-subject-out cross-validation |
| 8 | Fayad et al. 2021 [51] | 2021 | PD | - Diagnosis | Italian | - Voluntary PD - 51 PD - 51 healthy | - sustain vowel /e/ - 3 Italian sentences | - INTERSPEECH 2016 Computational Paralinguistics Challenge (ComParE 2016) audio feature set | - PD - Healthy | **Classification**   - Naive-Bayes (NB) - Support Vector Machine using Sequential Minimal Optimization (SVM-SMO) - Multi-Layer Perceptron (MLP)   10-fold CV |
| 9 | Rahman et al. 2021 [52] | 2021 | PD | - Diagnosis | USA | - Multiple locations over web - 262 PD - 464 healthy | - a popular pangram containing all the letters in the English alphabet- “the quick brown fox jumps over the lazy dog.” | - Pitch - Jitter - Shimmer - mel-frequency cepstral coefficients - HNR - Pitch estimation uncertainty - Measure of stochastic self-similarity in turbulent noise - Measure of the inability of maintaining a constant pitch - Deep learning–based PASE embeddings | - PD - Healthy | **Classification**   - Support Vector Machine - eXtreme Gradient Boosting (XGBoost) - Light Gradient-Boosting Machine (LightGBM) - Random Forest (RF) - DL feature embeddings + ML - Traditional feature + ML - Model interpretation with SHAP   leave-one-out validation |
| 10 | Cordella et al. 2021 [53] | 2021 | PD | - Diagnosis, Screening | Italy | - Single centre - 33 PD - 18 healthy | - 4 different sounds /ah/, /eh/, /iuh/ and /iamh/ | - Standard measures: fundamental frequency, jitter, shimmer, HNR - Nonstandard measures: Recurrence Period Density Entropy (RPDE), Signal Fractal Scaling Exponent (DFA), Pitch Period Entropy (PPE). - Cepstral measures: Cepstral Peak Prominence Smooth (CPPS), Mel Frequency Cepstral Coefficients (MFCC) |  | **Classification**   - Support Vector Machine (SVM) - Artificial Neural Network (ANN) - K-Nearest Neighbor (KNN   10-fold CV |
| 11 | Vasquez-Correa et al. 2021 [54] | 2021 | PD | - Diagnosis | Spanish/Colombia | - Multiple locations - High quality dataset - 106 PD - 105 healthy - 94 PD with MDS-UPDRS-III) - 93 PD and 48 HC with m-FDA scored. - App based dataset - 38 PD - 60 healthy | - six diadochokinetic (DDK) exercises - reading of 10 sentences - a read text - a monologue where the participants were asked to speak about their daily routine - a monologue based on the description of the cookie theft picture | - Mel spectrograms | - PD - Healthy - Motor symtoms severity based on MDS-UPDRSIII - dysarthria severity based on the m-FDA scale | **Classification**   - Convolution Neural Network (ResNet18 architecture)   10-fold stratified cross-validation |
| 12 | Majda-Zdancewicz et al. 2021 [55] | 2021 | PD | - Diagnosis | Poland | - Single centre - 22 PD (14 women and 8 men) - 22 healthy (14 men and 8 women) | - the vowel \|a\| | - Cepstral coefficients - MFCC-based features - LFCC-based features - GTCC-based features - Spectrograms | - PD - Healthy | **Classification**   - Support Vector Machine (SVM) - Convolution Neural Network (AlexNet)   11-fold cross-validation |
| 13 | Quan et al. 2021 [56] | 2021 | PD | - Diagnosis | - Mandarin | - Single center - 30 PD - 15 healthy | - monophonic /a/ - a short sentence | - Linear scaled short-time Fourier transform (STFT) spectrogram - Mel-scaled STFT spectrogram - Constant-Q transform (CQT) spectrogram. - dynamic time-series Articulation Features (AFs) - BBEs (Bark band energies) - MFCCs (Mel Frequency Cepstral Coefﬁcients | - PD - Healthy | **Statistical analysis**   - Paired t-test to compare speech features between PD and healthy   **Classification**   - Decision Tree (DT) - Multiplayer Perceptron (MLP) - K-Nearest Neighbour (kNN) - Gaussian Naïve Bayes (GNB) - Support Vector Machine (SVM) - Bidirectional Long-short Term Memory Network (Bi-LSTM) - Convolution Neural Network (CNN)   10-fold CV and train-test split validation |
| 14 | Amato et al. 2021 [57] | 2021 | PD | - Diagnosis | Italy | - 2 recording conditions   Recording in a controlled environment   - 28 PD - 22 healthy   Recording in a home setting   - 26 PD - 18 healthy | - reading of a phonetically balanced text - execution of the syllables /pa/ and /ta/ - phonation of the vowels /a/, /e/, /i/, /o/, /u/ - reading of a list of phonetically balanced words - reading of a list of phonetically balanced sentences. | - Relative Spectral - Perceptual Linear Prediction (RASTA-PLP) - Spectral moments: Energy Transition Slope (ETS) - Mel Frequency Cepstral Coefﬁcients (MFCC) - Detrended ﬂuctuation analysis (DFA) - Intensity difference - Duration ratio | - PD - Healthy | **Classification**   - Naive Bayes (NB) - k-Nearest Neighbour (KNN) - Support Vector Machine (SVM) - Random Forest (RF) - Adaptive Boosting (ADA), Gradient Boosting (GB), and Bagging Ensemble (BAG) classiﬁers.   10-fold CV |
| 15 | Tandjung et al. 2021 [58] | 2021 | PD | - Diagnosis | Taipei | - Single centre - 145 PD patient recordings - 55 PD controls who were patients with suspected PD but not PD after clinical assessment - 55 healthy recordings | - vocal /a/ and vocal /u/ | - fundamental frequency, Shimmer, and jitter - noise-related measurements (NHR) - voice irregularity related measurements - sub-harmonic and components-related measurements - voice break-related measurements | - PD - PD control - Healthy | **Classification**   - Tabular model of FastAI   Train-test validation |
| 16 | Jeancolas et al. 2021 [59] | 2021 | PD | - Early detection | French | - Single center - 121 PD - 151 healthy - High-quality records - 115 PD (74 males, 41 females) - 91 HC (48 males, 42 females) - Telephone recording - 101 PD (63 males, 38 females) - 61 HC (36 males, 25 females) | - Reading (1 min) - sentence repetition - free speech (participants were asked to talk about their day during 1 min) - fast syllable repetitions/ diadochokinesia (DDK) tasks | - X-vector speech embeddings based on MFCC - Energy and MFCC-based features | - PD (diagnosed less than four years before) - Healthy | **Classification**   - Multi-dimensional MFCC-GMM models and ensembles - For telephone recordings: X-vectors with Deep Neural Network (DNN) SRE16 - For high-quality recordings: X-vectors with voxceleb model - Classification based on similarity measures with respect to trained X-vectors   repeated random subsampling cross-validation |
| 17 | Goyal et al. 2021 [60] | 2021 | PD | - Diagnosis | English | - Single centre collected, multiple ethnicity with public data   Public dataset (Mobile Device Voice Recordings at King’s College London (MDVR-KCL)   - 16 PD - 21 healthy   Collected   - 12 healthy (from India) | - 2 reading paragraphs | - Time frequency-based information of high resonating components of voice signals converted into Power Spectral Density (PSD) | - PD - Healthy | **Classification**   - Convolution Neural Network (CNN) - k-Nearest Neighbour (KNN) - Support Vector Machine (SVM)   Train-validation splits |
| 18 | Carrón et al. 2021 [61] | 2021 | PD | - Diagnosis | English | - Single centre collected, multiple centers with public data   Collected dataset   - 30 PD (24 men, 6 women) - 30 healthy (26 men, 4 women)   Public dataset (mPower study)   - 30 PD (24 men, 6 women) - 30 healthy (26 men, 4 women) | - sustained phonations of /a/ vowel | - Jitter, Shimmer - CPP - HNR - glottal-to-noise excitation ratio - zero crossing rate - 3 GQ features - MFCCs (13 features) - correlation dimension - RPDE - pitch period entropy - Hurst’s exponent - LZ-2 - permutation entropy - Shannon’s entropy - first minimum in mutual information - MFSW - first zero in correlation function | - PD - Healthy | **Classification**   - Passive Aggressive - Perceptron - Support Vector Machine (SVM)) - Logistic Regression (LR) - Random Forest (RF) - Gradient Boosting   5-fold CV |
| 19 | Ali et al. 2021 [62] | 2021 | PD | - Diagnosis | Lithuanian | Not available | - phonation: vowel “a,” - speech: a phonetically balanced sentence in Lithuanian language | - Voiced/unvoiced segments - 17 set of feature sets | - PD - Healthy | **Classification**   - Linear Discriminant Analysis (LDA) - Gaussian Naive Bayes (GNB) - K-Nearest Neighbours (KNN) - Support Vector Machine (SVM) - Artiﬁcial Neural Network (ANN)   Train-test hold out validation |
| 20 | Zhang et al. 2020 [63] | 2020 | PD | - Diagnosis - severity assessment | Chinese | - single center, multiple ethnicity using public data - 3 subsets of data from a public dataset from the UCI repository - D1: 23 PD, 8 HC - D2: 48 PD, 20 HC - D3: 42 PD, 0 HC - Collected dataset (in China) - 14 PD - 30 Healthy | - five long vowels with the following English phonetic symbols: [ɑ:], [ :], [i:], [ :], and [u:]; | Linear features   - Pitch based - Jitter, shimmer based - Harmonics-to-noise ratio (HNR) and noise-to-harmonics ratio (NHR)   Non-linear features   - Detrended fluctuation analysis - Recurrence period density entropy - Correlation dimension - Pitch period entropy | - PD - Healthy - UPDRS score | **Classification**   - Support Vector Machine (SVM) - Artiﬁcial Neural Network (ANN) - Naive Bayes (NB) - Logistic Regression (LR)   **Regression**   - Support Vector Regression (SVR) - Linear Regression - LASSO regression.   5-fold CV |
| 21 | Altay 2020 [64] | 2020 | PD | - Diagnosis | Istanbul | - Single centre - 188 PD - 64 healthy | - phonation of the vowel /a/ | - Wavelet Transform based Features - Time-Frequency Features - TWQT Features - MFCC - Vocal Fold Features | - PD - Healthy | **Classification**   - AI-based multi-objective Association rule mining (MARM) with MOPNAR, NICGAR, QAR_CIP_NSGAII algorithms |
| 22 | Vásquez-Correa et al. 2019 [65] | 2019 | PD | - Diagnosis | Colombian Spanish native speakers | Multiple ethnicities using public data  Collected   - 44 PD - 40 healthy   public databases   - PC-GITA (Spanish) : 50 PD - German data 88 PD patients and 88 HC subjects - Czech: 20 PD patients and 15 HC subjects | - 6 DDK exercises: the rapid repetition of the syllables /pa-ta-ka/, /pe-ta-ka/, /pa-kata/, /pa/, /ta/, and /ka/. - read sentences - a read story of 36 words - a monologue. | - Time-frequency representations (TFR) - Extended Geneva minimalistic acoustic parameter set (EGeMAPS) | - PD: initial, intermediate and higher states as per MDS-UPDRS-III sub scores - Healthy | **Classification**   - Support Vector Machine (SVM) - Convolution Neural Network (CNN) - CNN + SVM (to integrate 3 modalities)   Repeated Train-validation-test procedure |
| 23 | Camnos-Roca et al. 2018 [66] | 2018 | PD | - Diagnosis | Spanish | - Single ethnicity - 40 PD - 40 healthy | - sustain phonation of /a/ | - perturbation measures: jitter, shimmer - Signal-to-noise ratio (SNR) measures: - Harmonic-to-noise ratio (HNR) - Glottal-to-Noise Excitation (GNE). - Mel Frequency Cepstral Coefﬁcients (MFCC) - nonlinear speech processing-based features: Recurrent Period Density Entropy (RPDE), Detrended Fluctuation Analysis (DFA), Pitch Period Entropy (PPE) [13], Correlation dimension (D2), and three entropy variants (permutation entropy, fuzzy entropy and sample entropy). | - PD - Healthy | **Classification**   - LASSO regression. - Ridge regression - Enet regression   10-fold CV |
| 24 | Montaña et al. 2018 [67] | 2018 | PD | - Diagnosis, Early diagnosis | Spanish | - Single ethnicity - 27 PD - 27 healthy | - steady /pa/-/ta/-/ka/ syllable | - Temporal features on VOT segments: time duration-based features, VOT ratio, CVRatio, Vowel Variability Quotient (VVQ), Consonant Variability Quotient (CVQ) and articulation rate - Spectral features on VOT segments: MFCC-based features and spectral moments based on linear predictive coding | - PD (mild or mild stages in the H&Y scale) - Healthy | **Classification**   - Support Vector Machine (SVM)   10-fold CV and Leave-one-out validation |
| 25 | Vaiciukynas et al. 2017 [68] | 2017 | PD | - Diagnosis | - Lithuanian | - Single center - 64 PD - 35 Healthy | - the vowel /a/ voicing task - a short sentence in Lithuanian language | - 12 OpenSMILE features - Essentia descriptors - MPEG7 descriptors - KTU features - jAudio features - YAAFE features - Tsanas features | - PD - Healthy | **Classification**   - Random Forest (RF)   out-of-bag (OOB) validation |
| 26 | Sztaho et al. 2017 [69] | 2017 | PD | - Diagnosis - Severity assessment | - Hungarian | - Single ethnicity - 51 PD - 27 Healthy | - a read text - a monologue | - Consonants and vowels durations, intervals - Speech durations - Articulation rate - Phonemes duration - pauses | - PD - Healthy - PD Severity as per HY scale | **Classification**   - k-Nearest Neighbours (KNN) - Support Vector Machines (SVM) - Artificial Neural Network (ANN) - Deep Neural Network (DNN) - Ensemble approach   **Regression**   - Linear Regression - Support Vector Regression (SVR) - Artificial Neural Network (ANN) - Deep Neural Network (DNN)   leave-one-out cross validation |
| 27 | Orozco-Arroyave et al. 2013 [70] | 2013 | PD | - Diagnosis | - Spanish | - Single centre - 50 PD - 50 healthy | - 5 Spanish vowels | - Linear prediction coeﬃcients - (LPC), - Linear prediction cepstral Coeﬃcients (LPCC) - Mel-frequency cepstral coeﬃcients (MFCC), perceptual linear prediction coeﬃcients (PLP) - Relative spectra coeﬃcients (RASTA) | - PD - Healthy | **Classification**   - 2-stage Support Vector Machine (SVM) |
| 28 | Sakar et al. 2013 [71] | 2013 | PD | - Diagnosis | - Turkish | - 20 PD - 20 healthy individuals | - Train - Numbers from 1 to 10, four rhymed sentences, nine words in the Turkish language along with sustained vowels “a”, “o”, and “u.” - Test - sustained vowels “a” and “o” | - Jitter - Shimmer - Pulse parameters based on period and pulses. - Pitch-based parameters - Harmonicity parameters: SNR, HNR, Autocorrelation | - PD - Healthy | **Classification**   - k-Nearest Neighbours (KNN) - Support Vector Machines (SVM)   leave-one-subject-out |
| 29 | Viswanathan et al. 2019 [72] | 2019 | PD | - Diagnosis | - Australia | - Single centre - 24 PD - 22 Healthy | - three sustained phonetic sounds /a/ (as in car), /u/ (as in wool) and /m/ (as in mum). | - Normalized mutual information obtained through probability distributions | - PD - Healthy | **Statistical analysis**   - Descriptive statistics between 2 groups - One-way ANOVA test to show the statistical difference of the features between PD and control subjects.   Pearson correlation coefficient |
| 30 | Zhang et al. 2017 [73] | 2017 | PD | - Diagnosis | - Mandarin | - Single ethnicity - 16 PD - 20 Healthy | - vowel \|a\| | - Perturbation features: fundamental frequency, jitter, shimmer - Energy distribution features: Noise to Harmonic ration, Harmonic to noise ratio, glottal-to-noise excitation (GNE), Empirical mode decomposition excitation ratio (EMD-ER) - Non-linear features: correlation dimension, permutation entropy and detrended fluctuation analysis, recurrence period density entropy | - PD - Healthy | **Statistical analysis**   - 2-way repeated measure ANOVA with post hoc analysis - 2 independent sample t-tests (for normally distributed features)   Mann-Whitney rank sum test for features without normal distribution) |
| 31 | Vizza et al. 2019 [74] | 2019 | PD and Multiple Sclerosis | - Diagnosis, - Differential diagnosis | - Italy | - Multiple centres - 60 PD - 54 Multiple Sclerosis (MS) - 39 healthy | - sustained vowel (/a/, /e/, /i/, /u/) phonation | - Fundamental frequency - Jitter, shimmer - Noise-to-Harmonic Ratio (NHR) - Vowel metrics: Vowel Space Area (VSA), triangular VSA (tVSA), the quadrilateral VSA qVSA) - Formant Centralization Ratio (FCR) | - Healthy - PD - MS | **Statistical analysis**  Analysis of variance (ANOVA) to measure changes among the three classes and among all the extracted features. |
| 32 | Viswanathan 2021 [75] | 2021 | PD | - Diagnosis - severity assessment | - Australia | - volunteered PD, single centre - 26 PD - 22 healthy | - 3 sustained phonetic tasks (/a/, /u/, /m/) | - Glottic Cycle features - Normalised amplitude quotient (NAQ) - Amplitude quotient (AQ) - Closing quotient (ClQ) - Primary and secondary open quotients (OQ1,OQ2) - Amplitude-based open quotient (OQa) - Quasi open quotient (QOQ) - Primary and secondary speed quotients (SQ1,SQ2) - Temporal and Spectral Features based on : Harmonics - Formants, MFCC, Jitter, Shimmer, HNR | - PD - Healthy - UPDRS score on and off state | **Statistical analysis**   - Spearman correlation to assess the relationship between UPDRS score and voice features   **Regression**   - Support Vector Machine regression (SVR) - Random Forest regression   AdaBoost regression |
| 33 | Das et al. 2019 [76] | 2019 | Atypical Parkinsonian Syndromes (APS), s (Progressive Supranuclear Palsy (PSP) and Multiple System Atrophy (MSA) | - Differential Diagnosis | - France | - Longitudinal collection - 12 probable PSP - 13 probable MSA | - sustained phonation of the vowel /a/ - fast /pa/-/ta/-/ka/ syllable repetition - a reading passage - a monologue on a given topic | Hypokinetic dysarthria   - Jitter, Shimmer, Harmonics to-noise ratio (HNR), pace acceleration, the percentage of pause time relative to total speech time, the average number of pauses per second, The intra-word pause ratio, pitch, and inﬂection changes   Spastic dysarthria   - fraction of pitch frames marked as unvoiced, Pitch ﬂuctuation   Ataxic dysarthria.   - frequency tremor intensity index (FTRI) | - Probable PSP - Probable MSA | **Statistical analysis**   - Univariate statistical analysis for feature selection   **Classification**   - Factorial Discriminant Analysis (FDA) - Gaussian classiﬁer   1D Linear Support Vector Machine (LSVM) |
| 34 | Li et al. 2018 [77] | 2018 | PD and APS | - Differential Diagnosis - progressive supranuclear palsy (PSP) and multiple system atrophy (MSA) |  | - Longitudinal study - 12 patients with probable PSP - 13 patients with probable MSA | - sustained phonation of the vowel /a/ - fast /pa/-/ta/-/ka/ syllable repetition - a monologue on a given topic | - Voicing features: Jitter, Shimmer, and HNR, the degree of voicelessness (DUV), the standard deviation of voice pitch (F0 SD), and the frequency tremor intensity index (FTRI). - Articulation measures: Alternating Motion rate (AMR) based features - Prosodic features: F0-based features, pause-based features (the average number of pauses per second, Percentage of pause time (PPT), The intra word pause ratio | - PSP - MSA | **Classification**   - Factorial Discriminant Analysis (FDA) + Linear Support Vector Machine (SVM) - FDA + Logistic Regression   Leave-One-Speaker-Out validation |
| 35 | Hemmerling and Wojcik-Pedziwiatr 2022 [78] | 2022 | PD | - Treatment monitoring - Severity assessment | - Poland | - Single center - 27 PD patients at different time points of treatment | - sustained vowels: /a/, /e/, /i/, /o/ and /u/. | - Phonatory features: fundamental frequency, jitter and shimmer coefﬁcients, energy, 0-, 1-, 2-, 3- spectral moment, power spectral moment, kurtosis and curvature - Articulatory features: the 1st, 2nd, and 3rd formant frequency - Additional features: MFCCs   , Positive and negative amplitude-based features, Perceptual linear prediction (PLP) coefﬁcients based features | - UDPRS scores after 30, 60 and 120 and 180 minutes of levodopa medication. - after more than 3 hours of medication when patients reported recurrence of mitigated symptoms | **Statistical analysis**   - Pearson (r) correlation to detect linear relationships. - Spearman (r) correlation to detect non-linear relationships.   **Regression**   - Multiple Linear Regression (MLR) - Support Vector Regression (SVR) - Random Forest Regression   10-fold CV |
| 36 | Tunc et al. 2020 [79] | 2020 | PD | - Severity assessment | - Istanbul | - Multiple cohorts using public datasets   Collected dataset   - 86 PD patients (49 male and 37 female   Public dataset   - Parkinson’s telemonitoring dataset - 2 early-stage PD patients. | - phonation of the vowel /a/ | Linear processing-based features   - jitter, shimmer, fundamental frequency (F0), harmonics-to-noise ratio (HNR), noise to-harmonics ratio (NHR), intensity, formant frequencies, bandwidths [42, 86], root mean square (RMS) energy, the strength of excitation (SoE) [46], and cepstral peak prominence (CPP) measures   Other techniques-based features   - Recurrence period density entropy (RPDE) - Detrended fluctuation analysis (DFA) - Pitch period entropy (PPE) - Mel frequency cepstral coefficients (MFCCs) - Wavelet transform (WT) features related with F0 - Glottis quotient (GQ) - Glottal to noise excitation (GNE) - Vocal fold excitation ratio (VFER) - Empirical mode decomposition (EMD) - TQWT-based features | - motor-UPDRS score - total-UPDRS scores. | **Statistical analysis**   - Correlation of UPDRS scores and speech features   **Regression**   - XGBoost implementation of the gradient boosting decision tree (GBDT)   Leave-one-subject-out CV |
| 37 | Vandana et al. 2021 [80] | 2021 | PD | - Treatment monitoring (on voice) | - India | - Single centre - 25 PD - 25 Healthy | - sustained vowel /a/ | - Fundamental frequency (F0) - Jitter - Shimmer - frequency tremor intensity index - soft phonation index (SPI). - Variation in amplitude, frequency - Voice Handicap Index (VHI) | - PD patients on and off states of medication - Motor impairment scores | **Statistical analysis**   - Descriptive statistics of acoustic features PD on/off states - Wilcoxon signed-rank test to compare acoustic parameters between PD on and off states.   Spearman’s rank correlation coefﬁcient between acoustic features and motor impairment scores |
| 38 | Jain et al. 2021 [81] | 2021 | PD | - Treatment monitoring | - German and English | - Single center - 16 PD patients (10 female, 6 male) | - Digits count to 10 (German) - name the months in a calendar year from January to December twice - The Vowels task: English vowels /a/-/e/-/i//o/-/u/ three times. - Pataka test: /pataka/, /pakata/, /petaka/, and /pekata/ - 3 German tongue twisters - Read short German text - Cookie Theft description. | - Phone attribute posterior probability-based features from MFCCs - Spectrograms | - PD before medication - PD after medication | **Statistical analysis**   - Two-sample paired t-test on phone attribute features at PD on/off states - feature wise correlation within PD on/off states   **Classification**   - Convolutional Recurrent Neural Networks (CRNN) - Instance based ML - Phone Attribute Codebooks (PAC)- instance-based machine learning   leave-one-out cross-validation |
| 39 | Gaballah et al. 2018 [82] | 2018 | PD | - Treatment monitoring (speech amplifiers) | - Canada | - Single centre - 11 PD - 10 healthy | - Sentence reading - Natural conversation | - Mel-frequency cepstral coefficients (MFCC) - Gammatone frequency cepstral coefficients (GFCCs) | - PD - Healthy | **Classification**  DNN regression (with optimizers) |
| 40 | Gaballah et al. 2019 [83] | 2019 | PD | - Treatment monitoring (speech assistive amplification devices), Speech Quality assessment |  | - Single centre - 11 PD - 10 healthy | - unscripted conversation in quiet and in the presence of background noise - reciting a given sentence in quiet and noisy environments | - CPP - Filter bank-Based Features - MFCCs - Gammatone frequency cepstral coefﬁcients (GFCCs) - Modulation-Based Features - The speech-to-reverberation masking ration (SRMR) - Modulation Area (ModA) parameter. - Linear Prediction based feature extraction methodology in Low Complexity Quality Assessment (LCQA) - spectral ﬂatness, the excitation variance, the signal variance, the spectral centroid, and the spectral dynamics | - PD - Healthy - Subjective voice quality scores | **Statistical analysis**   - ANOVA group analysis of subjective speech quality metrics between different groups eg, PD vs HC, noise vs quiet, free speech vs reading sentence - Feature mapping between multi-dimensional objective scores and subjective scores using 4 ML algorithms: LR (Linear Regression), SVR (Support Vector regression), GPR, and DNN (Deep Neural Network) and then conducting correlation analysis   Correlation between subjective scores and one-dimensional objective speech features |

## **Alzheimer’s Disease (AD) and cognitive disorders**

|  | **Study** | **Year** | **Disease** | **Clinical purpose** | **Country/Language** | **Sample size** | **Speech test** | **Speech features** | **Ground truth** | **Data science approach** |
| --- | --- | --- | --- | --- | --- | --- | --- | --- | --- | --- |
| 41 | Shimoda et al. 2021 [86] | 2021 | AD | - Diagnosis | Japan | - Multiple locations through volunteering - 99 healthy - 24 AD | - One-minute free talk- with an AI program | - Sounding and silent locations - Intensity - Pitch - center of gravity, skewness, kurtosis, and standard deviation of spectrum | - AD - Healthy | **Classification**   - Extreme Gradient Boosting (XGBoost) - Random Forest (RF) - Logistic Regression (LR)   Train-test validation |
| 42 | Toth et al. 2018 [87] | 2020 | MCI | - Diagnosis MCI | Hungarian | - Single ethnicity - 38 healthy controls - 48 MCI patients | - “immediate recall”): to talk about the events they saw in a film that was showed to the participant - “spontaneous speech”: describe their previous day - "delayed recall" : to talk about second film after a one-minute long distraction | - hesitation ratio - speech tempo - length and number of silent and filled pauses - length of utterance | - MCI - Healthy | **Statistical analysis**   - one-tailed t-test for group comparisons of temporal features   **Classification**   - Naive Bayes (NB) - Support Vector Machine (SVM) - Random Forests (RF)   Leave-one-out cross validation |
| 43 | Themistocleous et al. 2018 [88] | 2018 | MCI | - Diagnosis MCI | Sweden | - Single centre - 30 healthy controls - 25 MCI | - Read a short passage, consisting of 144 words, | - vowel duration - vowel formants (F1 to F5) - fundamental frequency | - MCI - Healthy | **Classification**   - Deep Neural Network Architectures based on MLP   5-fold CV and train-test split validation |
| 44 | Nagumo et al. 2020 [89] | 2020 | MCI | - Diagnosis MCI - Differential diagnosis | Japan | - Multiple cities - 6343 healthy controls - 1601 MCI- mild cognitive impairment - 367 GCI - global cognitive impairment - 468 MCI with GCI - mild cognitive impairment with global cognitive impairment (a combined status of mild cognitive impairment and global cognitive impairment), | - Vowel utterances - Tongue twister - Diadochokinetic - Short sentences | - Vowel utterance task -vowel formant frequency (F1 and F2), fundamental frequency (F0), the triangular vowel space area, vowel articulation index - Tongue twister and DDK task -utterance pauses, length of pauses, number of uttered syllables, duration, number of the mean and standard deviation of syllable duration, together with the mean and standard deviation of the power of maximum and minimum. - Short sentences - temporal features and mean utterance time ratio | - HC: MMSE of 2430, NCGG-FAT of 0 - MCI: MMSE of 24-30, NCGG-FAT of 1-4 - GCI: MMSE of 20-23, NCGG-FAT of 0 - MCI with GCI: MMSE of 20-23, NCGG-FAT of 1-4 | **Classification**   - Logistic Regression (LR)   Nested cross validation with stratified data split |
| 45 | Munthuli et al. 2021 [90] | 2021 | AD, MCI, Normal cognition | - Diagnosis - Differential diagnosis | Thailand | - Single ethnicity - 30 Normal cognition (23 female, 7 males) - 30 MCI (17 females, 13 males) - 30 AD (17 females. 13 males) | - 16 speech-related tasks including a picture description task, language reading task | - Emobase feature set | - Healthy - MCI - AD | **Classification**   - Linear Discriminant Analysis (LDA) - Support Vector Classification (SVC) - Logistic Regression (LR) - Adaptive Boosting (AdaBoost) - Random Forest (RF) - Multi-Layer Perceptron (MLP)   5-fold CV |
| 46 | Mirzaei et al. 2018) [91] | 2018 | Early AD, MCI, Healthy | - Diagnosis (early) - Differential diagnosis | France | - Single centre - 16 Early Stage of Alzheimer’s Disease (ES-AD) - 16 MCI - 16 healthy | - read sentences of a French book | - Prosodic features based on fundamental frequency, - Temporal parameters based on speech and voiced segments. - Quality measures based on jitter, shimmer, and Harmonics-Noise-Ratio (HNR). - Mel-Frequency Cepstral Coefficients (MFCCs) and Filter Banks Energies (FBEs) | - Early AD - Healthy - MCI | **Classification**   - K-Nearest Neighbour (KNN) - Support Vector Machine (SVM) - Decision Tree (DT).   8-fold CV |
| 47 | König et al. 2015 [92] | 2015 | AD, MCI, Healthy | - Diagnosis - Differential diagnosis | France | - Singel centre - 15 HC - 26 AD - 23 MCI | - a counting backward task - a sentence repeating task - an image description task - a verbal ﬂuency task | - Countdown and picture description tasks- : features based on length of voice segment, silence segment, periodic segment, aperiodic segment - Sentence repeating task: Vocal reaction time, relative length, silence, insertions, deletions, and irregularity-based features - Semantic fluency task: intensity and periodicity, distance between word position features | - HC - MCI - AD | **Statistical analysis**   - Intergroup comparison of continuous variables with Mann-Whitney U test   **Classification**   - Support Vector Machine (SVM)   Random sub-sampling (train-test) cross validation |
| 48 | Bertini et al. 2022 [93] | 2022 | Early dementia, MCI | - Diagnosis - Differential diagnosis | Italy | - Single centre - 48 healthy - 48 cognitively impaired subjects (32 MCI and 16 early dementia) | - Spontaneous speech induced by 3 questions ( on a picture, about a working day, about a dream) | - Mel spectrograms | - Healthy - MCI - Early dementia | **Classification**   - Autoencoder-based recurrent neural network (AuDeep) + Multi-Layer Perceptron (MLP) - DepAudioNet based on CNN and LSTM (re-implementation)   20-fold CV |
| 49 | Yamada et al. 2022 [94] | 2022 | AD and dementia with Lewy bodies (DLB) | - Diagnosis - Differential Diagnosis | Japan | - Multi-centre through volunteers - 45 AD - 27 DLB - 49 healthy (cognitive normal) | - counting backwards, - subtraction - phonemic verbal fluency task - semantic verbal fluency task - picture description with the Cookie Theft picture from the Boston Diagnostic Aphasia Examination. | - Prosodic features based on pause duration, pitch variation, the phoneme rate. - acoustic features based on jitter and shimmer. - MFCCs - Linguistic features based on transcriptions | - AD - DLB - CN | **Statistical analysis**   - Group differences between CN, AD, and DLB with the chi-square test (for categorical data) and one-way analysis of variance (ANOVA) (for continuous data) - one-way analyses of covariance (ANCOVAs) to compare speech features between groups after controlling the use of medication - A two-way ANOVA to examine the effects of the dementia type (AD, DLB) and disease stage (MCI, dementia) on the speech features.   **Classification**   - Support Vector Machine (SVM)   leave-two-subjects-out cross-validation |
| 50 | Sumali et al. 2020 [95] | 2020 | Dementia and pseudo dementia | - Differential diagnosis | Japan | - Single centre, different cohorts - Train set (age matched) - 24 Depression - 29 Dementia - Test set (young depression, old dementia) - 53 depression - 14 dementia | - a typical clinical interview concerning the patient’s daily life and mood encouraging free talk | - Pitch, - harmonics-to-noise ratio (HNR) - zero-crossing rate (ZCR) - Mel-frequency cepstral coefficients (MFCC) - Gammatone cepstral coefficients (GTCC) - mean and median frequency - signal energy - spectral centroid - spectral roll off point | - HAMD score - MMSE score | **Statistical analysis**   - Correlation   **Unsupervised learning**   - K-mean clustering   **Classification**   - Support Vector Machine (SVM) with LASSO for feature selection   Nested 10-fold CV |
| 51 | Al-Hameed et al. 2019 [96] | 2019 | Cognitive disorders | - Differential diagnosis | UK | - Singel center - 15 ND (neurodegenerative disorders- 10 cases of AD, 2 amnestic MCI, 2 Bv FTD and one vascular dementia - 15 FMD (Functional Memory Disorder) | - conversational activities with neurologists | - Speech and silent statistics - Phonation and voice quality features: Fundamental frequency (F0), Harmonic-to-noise ratio (HNR), Noise-to-harmonic ratio (NHR), shimmer, jitter, voice break based features   Spectral features   - Mel frequency cepstral coefficients (MFCC) - Filter bank energy coefficient (Fbank) - Spectral Subband Centroid (SSC) | - ND cognitive disorder - FMD cognitive disorder | **Classification**   - Support Vector Machine (SVM) - Stochastic Gradient Descent (SGD) - Random Forest (RF) - Adaboost - Multi-Layer Perceptron (MLP)   Nested- 5-fold CV |
| 52 | König et al. 2021 [97] | 2021 | neuropsychiatric symptoms (NPS) | - Severity assessment | France | - Singel centre - 141 MCI-mild neurocognitive disorder (92 females and 49 males) | - talk about a positive event in their life - talk about a negative event in their life. | - Temporal features: speech ratio, Speech interval, total phonation time, Number of pauses - Source features: Harmonics to noise ratio, Sound to noise ratio, Jitter, zero crossing - Amplitude-based features - Power-based features - Prosodic features based on fundamental frequency. - Spectral features on MFCC, Frequency, and Power spectrum | - NPI score (depression, anxiety, apathy) | **Statistical analysis**   - Correlation between NPI scores and speech features   **Regression**   - Support vector regression (SVR) - Lasso (Linear Regression with L1 regularization).   Leave-One-Out Cross-Validation |

## **Multiple Sclerosis (MS)**

|  | **Study** | **Year** | **Disease** | **Clinical purpose** | **Country/Language** | **Population Characteristics** | **Speech task** | **Speech features** | **Ground truth** | **Data science approach** |
| --- | --- | --- | --- | --- | --- | --- | --- | --- | --- | --- |
| 53 | Fazeli et al. 2018b [99] | 2018 | MS | - Diagnosis - Severity Assessment | Iran | - Random sample from a society - 47 MS - 20 healthy | - Sustain the vowel /a/ - Three words with CVC syllable structure (dad, dud, did) | - Dysphonia Severity Index (DSI) based on Maximum phonation time, Jitter, maximum fundamental frequency, and minimum intensity - Formant Centralization Ratio (FCR) based on the first 2 formants (F1, F2) of vowels | - MS vs healthy - Diseased duration - Disease severity based on the Expanded Disability Status Scale (EDSS) | **Statistical analysis**   - Statistical comparison of DSI and FCR between MS and healthy groups (independent t-test and Mann-Whitney U test) - Spearman Correlation between speech parameters (FCR, DSI) and neurological status   **Classification**   - Logistic regression between MS and healthy groups   **Regression**   - Linear Regression between FCR and disease severity |
| 54 | Fazeli et al. 2018a [100] | 2018 | MS | - Diagnosis | Iran | - Sample from a MS society - 40 MS - 20 healthy | - phonate vowel /a/ | - Dysphonia Severity Index (DSI) based on Maximum phonation time, Jitter, maximum fundamental frequency, and minimum intensity | - MS vs healthy | **Statistical analysis**   - Statistical comparison of DSI between MS and healthy groups (independent t) |
| 55 | Svoboda et al. 2022 [101] | 2022 | MS | - Diagnosis | Germany | - Single ethnicity, single centre - 65 MS - 64 Healthy | - 230 syllables long Crech text | - Speech duration - Silence-to-speech ratio - Vowel-to-speech ratio - Cumulative Slope Index (CSI) of vowel duration - CSI of fundamental frequency - Quantile difference of fundamental frequency - Unvoiced stop mean duration - CSI of intensity - Standard deviation of the spectral centroid of/s/ - Standard deviations of F1, F2 and F3 - Demographic variables: Age, gender | - MS vs healthy | **Statistical analysis**   - Univariate statistical significance of features with the Kolmogorov-Smirnoff statistics - Multivariate statistical significance of features using generalized linear regression   **Classification**   - eXtreme Gradient Boosting (XGBoost) - Generalized regression Boosting Model (GBM) - Generalized Linear Model (GLMnet) - k-Nearest Neighbours (KNN) - Multi-Layer Perceptron Neural Network (MLP-NN) - Random Forrest (RF) - Support Vector Machine (SVM)   5-fold CV with train-test split |
| 56 | Gosztolya et al. 2022 [102] | 2022 | MS | - Diagnosis | Hungary | - 2 collection centres - 22 MS - 19 healthy | - To talk about on previous day - Summary of a story heard (narrative recall). - read aloud several speciﬁc non-words (CVCV sequences). | - 40 Mel-frequency ﬁlter banks along with raw energy along with delta and delta of delta | - MS vs Healthy | **Classification**   - Deep Neural Network (DNN) as a feature extractor + SVM classification   Nested 1-fold CV |
| 57 | Vizza et al. 2017 [103] | 2017 | MS | - Diagnosis - Severity assessment | Italy | - Single centre - 7 Healthy - 18 Secondary Progressive Multiple Sclerosis (SPMS) - 35 Relapsing-Remitting Multiple Sclerosis (RRMS) | - sustained pronunciation of the ﬁve vowels /a/, /e/, /i/, /o/, /u/ | - Fundamental frequency (F0) - Jitter - Shimmer - Harmonic to Noise Ratio (HNR). - Vowel space area from first 2 formats (F1, F2) | - MS vs Healthy - MS stage: SPMS, RRMS | **Statistical analysis**   - Descriptive statistics of HS, SPMS, and RRMS groups - Statistical comparison of MS and healthy groups via t-test   **Visualizations**  Vowel metric analysis and graphical illustrations |

## **Amyotrophic Lateral Sclerosis (ALS)**

|  | **Study** | **Year** | **Disease** | **Clinical purpose** | **Country/language** | **Population Characteristics** | **Speech task** | **Speech features** | **Ground truth** | **Data science approach** |
| --- | --- | --- | --- | --- | --- | --- | --- | --- | --- | --- |
| 58 | Tena et al. 2021 [105] | 2021 | ALS | - Diagnosis - Detect bulbar involvement | Spanish | - 45 ALS (14 with bulbar involvement) - 18 healthy | - five Spanish vowel segments (a, e, i, o, and u) | - jitter, shimmer, HNR, and pitch | - ALS with bulbar involvement - ALS without bulbar involvement - healthy | **Dimensionality reduction analysis**   - Principal Component Analysis for feature importance analysis   **Classification**   - Support Vector Machine (SVM) - Neural Networks - Linear Discriminative Analysis (LDA) - Logistic Regression (LR) - Random Forest (RF) - naïve Bayes (NaB),   Repeated 10-fold CV |
| 59 | Illa et al. 2018 [106] | 2021 | ALS | - Diagnosis | India | - Singel centre - 8 ALS with bulbar involvement - 8 healthy | - rehearsed speech : 2 sentences - spontaneous speech: a monologue to elicit a natural speech output. - repetition of words : 9 kannada words | - MFCC-based features | - ALS - Healthy | **Classification**   - Deep Neural Networks (DNN) - Support Vector Machines (SVM)   4-fold CV |
| 60 | Likhachov et al. 2021 [107] | 2021 | ALS | - Diagnosis | Belarus | - Single centre - 33 healthy - 31 patients with ALS with signs of bulbar involvement | - long pronunciation of the vowel sound / a / | - Jitter, shimmer - Based on F0-contour: pitch periods entropy (PPE), the pathology vibrato index (PVI) - Noise: HNR, GNE | - ALS - Healthy | **Classification**   - Linear discriminant analysis (LDA). - k-Nearest Neighbours (KNN)   9-fold CV |
| 61 | Mallela et al. 2020 [108] | 2020 | PD and Amyotrophic Lateral Sclerosis | - Diagnosis - Differential Diagnosis | 6 Different native speakers (Bengali, Hindi, Odiya, Tamil, Telugu, Kannada) | - Single centre - 60 ALS with different speech function severities - 60 PD - 60 Healthy | - Spontaneous speech (SPON) – 2 Talks in native language - Diadochokinetic rate (DIDK)- AMR: “pa-pa-pa”, “tata-ta”, “ka-ka-ka”, SMR: “pataka” and “badaga” - Sustained phoneme production (PHON) - 5 vowels : /a/, /i/, /o/, /u/, /æ/, 3 fricatives : /s/, /sh/, and /f/. | - MFCC | - ALS - PD - Healthy | **Classification**   - Support Vector Machine (SVM) - Deep Neural Network (DNN) - Convolution Neural Network – Log Short Term Memory Networks (CNN-LSTM)   5-fold CV |

## **Mild Traumatic Brain Injury (mTBI)**

|  | **Study** | **Year** | **Disease** | **Clinical purpose** | **Country/language** | **Population Characteristics** | **Speech task** | **Speech features** | **Ground truth** | **Data science approach** |
| --- | --- | --- | --- | --- | --- | --- | --- | --- | --- | --- |
| 62 | Daudet et al. 2017 [110] | 2017 | mTBI | - Diagnosis | US | - Across multiple teams - 486 healthy - 95 concussed | - 7 speech tasks including reading set of words, reading sentences, DDK tasks (/Pa, /Ka, /pa-ta-ka | - Temporal features: based on durations, pauses, DDK rates, DDK periods - Frequency-based features: based on pitch and power | - Healthy - concussed | **Classification**   - Logistic Regression (LR) |
| 63 | Falcone et al. 2013 [111] | 2013 | mTBI | - Diagnosis | US | - Across multiple teams - 105 baseline healthy athletics - 101 post healthy - 7 post mTBI | - spoke digit words in a given order | - pitch, formant frequencies F1-F4, jitter, shimmer, and harmonics-to-noise ratio (HNR). | - Baseline Healthy - Post healthy - Post mTBI | **Classification**   - One-Class Support Vector Machine (SVM) |
| 64 | Wall et al. 2022 [112] | 2022 | mTBI | - Diagnosis | UK | - Single team - 46 baseline healthy athletics - 7 post season concussed - 39 post-season healthy | - SCAT5 reading paragraph | - MFCC | - Baseline healthy - Post season concussed - Post-season healthy | **Classification**   - Particle swarm optimized (PSO) bidirectional long short-term memory attention (Bi-LSTM-A)   Train-validation-test split validation |

## **Huntington’s Disease (HD)**

|  | **Study** | **Year** | **Disease** | **Clinical purpose** | **Country/Language** | **Population Characteristics** | **Speech task** | **Speech features** | **Ground truth** | **Data science approach** |
| --- | --- | --- | --- | --- | --- | --- | --- | --- | --- | --- |
| 65 | Riad et al. 2022 [113] | 2022 | HD | - Severity assessment | French | - Multiple centres - 16 PreHD - 87 manifest/HD participants | - count aloud numbers from 1 to 20 (forward counting) - count the numbers backwards from 20 to 1 | Articulatory and phonatory deficiencies   - pronunciations errors based features - fundamental error based features - Intensity of vocalization based features   Rhythm and temporal statistics   - Task duration - pronounced numbers based features - Phones per second - Silences based features   Sequence errors and perseverations   - pronounced numbers and the target sequence based features - Total number of pronounced numbers - Total number of pronounced phones   Collateral track additions   - Involuntary/Abnormal vocalizations - filled pauses based features | - UHDRS total score - Functional score - Motor score - Cognitive score | **Regression**   - auto-machine-learning system, auto-sklearn   train-test cohorts |
| 66 | Gallezot et al. 2022 [114] | 2022 | HD | - Emotion recognition | French | - Multiple cohorts - 68 HD - 22 participants carrying the mutant HD gene without any motor symptoms (pre-manifest HD) - 25 healthy | - Interviews by neuropsychologists to complete 4 tasks - Task 1 (neutral) to describe the latest 24 h - tasks 2, 3, and 4 to elicit emotions by telling a story making the participants sad, angry, and happy, - The speech tasks were separated by non-emotional speech | - the Extended Geneva Minimalistic Acoustic Parameter Set (eGeMAPS) | - 4 emotions (Anger, Sadness, Joy, Neural) from three groups of HD, PreHD and Healthy | **Classification**   - Random Forest   Repeated10-fold nested CV |

## **Autism Spectrum Disorder (ASD)**

|  | **Study** | **Year** | **Disease** | **Clinical purpose** | **Country/Language** | **Population Characteristics** | **Speech task** | **Speech features** | **Ground truth** | **Data science approach** |
| --- | --- | --- | --- | --- | --- | --- | --- | --- | --- | --- |
| 67 | MacFarlane et al. 2022 [116] | 2022 | ASD | - Diagnosis | USA | - Multiple clinics - 88 ASD - 70 non-ASD (ADHD and TD groups) | - Friends, Relationship, and Marriage Conversation task in ADOS | - Cepstrum, delta cepstrum, delta delta cepstrum, log spectral entropy, F0, jitter, shimmer, harmonic-to-noise ratio (HNR), H1H2, root mean square (RMS). | - ASD - Non-ASD | **Classification**   - Support Vector Machine (SVM)   leave-one subject-out cross validation |
| 68 | Eni et al. 2020 [117] | 2020 | ASD | - Diagnosis - Severity assessment | Israel | - Single centre - 56 ASD - 10 suspicious ASD but received other diagnoses (e.g., language or developmental delays) - 6 Typical development controls | - recordings of ADOS sessions | - Prosodic and acoustic features: Pitch (fundamental frequency), formants, spectral sloe, jitter, energy, Zero Crossing Rate (ZCR) based features. - Conversational features: Vocalization rate, duration, turn taking, number of segments, based features | - ADOS scores | **Statistical analysis**   - Statistical association between ADOS score and speech features via Pearson correlation coefficient.   **Regression**   - Deep Neural Network (DNN) - Convolution Neural Network (CNN) - Multiple Linear Regression (MLR) - Support Vector Regression (SVR)   5-fold CV |

## **Other Neurological Diseases**

|  | **Study** | **Year** | **Disease** | **Clinical purpose** | **Country/Language** | **Population Characteristics** | **Speech task** | **Speech features** | **Ground truth** | **Data science approach** |
| --- | --- | --- | --- | --- | --- | --- | --- | --- | --- | --- |
| 69 | Suppa et al. 2021 [119] | 2021 | Essential Tremor (ET) | - Diagnosis - Treatment monitoring | Italy | - Single centre - 58 ET - ET with voice tremor - ET without voice tremor - ET with treatments for tremor - ET without drug treatment (17, 4 men) - 74 health | - sustained emission of a close mid-front unrounded vowel /e/ | - the frequency tremor frequency - the frequency tremor intensity index - INTERSPEECH2016 Computational Paralinguistics Challenge (IS ComParE 2016) feature data set. | - ET with voice tremor (ETVT+) - ET without voice tremor (ETVT-) - ET with drug treatment at baseline - ET with drug treatment after 1 week | **Statistical analysis**   - The unpaired Student t-test to compare voice samples recorded in ETVT+ and ETVT−patients, - A paired Student t-test to compare the same measures in ETVT+ patients under and not under therapy.   **Classification**   - Support Vector Machine (SVM)   **Speech impairment prediction**   - Artificial Neural Network (ANN)   10-fold CV |
| 70 | König et al. 2019 [120] | 2019 | Apathy in neurocognitive disorders | - Diagnosis - Severity assessment | French speakers | - Single ethnicity - 30 Apathetic patients - 30 Non-apathetic patients - Both with mild to moderate neurocognitive disorders | - A talk about a positive event in the life - A talk about a negative event in the life | - Prosodic features- : fundamental frequency, periodicity - Formant: F1-F3, jitter - Source features: shimmer, HNR, sound segments, pause segments - Temporal features: duration, speech duration, pause duration, speech proportion, speech rate, articulation rate | - Apathetic neurocognitive disorder - Non-apathetic neurocognitive disorder | **Statistical analysis**   - Statistical group comparisons between non-apathetic and apathetic group using Kruskal-Wallis tests - Spearman correlation between acoustic features and Apathy Inventory (AI) sub-scales   **Classification**   - Logistic Regression (LR)   Leave-one-out cross validation |
| 71 | Aggarwal et al. 2021 [122] | 2021 | Intellectual disability | - Diagnosis | English/Hindi | - Single ethnicity - 24 typically developed (TD) children - 24 Intellectually Disabled (ID) children | - continuous phonation task - imitating instructor's voice | - Mel-Frequency Cepstral Coefﬁcients - linear predictive coefﬁcients (LPC, LPCC, and WLPCC) | - Intellectual Disability - Healthy | **Classification**   - Support Vector Machines (SVM) - Random Forest (RF) - Artiﬁcial Neural Networks (ANN)   10-fold CV with train-test split |
| 72 | Lauraitis et al. 2020 [123] | 2020 | central nervous system disorders (CNSD) | - Diagnosis | Lithuania (Lithuanian/English speech) | - Singel centre - 7 patients with early-stage CNSD (3 Huntington, 1 Parkinson, 1 cerebral palsy, 1 post-stroke, 1 early dementia) - 8 healthy | - read a short text of predeﬁned poems | - WST (wavelet time scattering, analytic Gabor) - cepstrum domain (pitch contours, MFCC, GTCC) - auditory spectrograms based features: spectral Slope, spectral Skewness, spectral Spread, spectral Decrease, spectral Kurtosis, spectral Flux, spectral Rolloff, spectral Flatness, spectral Entropy | - CNSD - Healthy | **Classification**   - Support Vector Machine (SVM) - Bidirectional recurrent neural network with Long short-term memory (Bi-LSTM)   Train-test split |

## **References**

44. *Wang Q, Fu Y, Shao B, Chang L, Ren K, Chen Z, Ling Y. Early detection of Parkinson's disease from multiple signal speech: based on Mandarin language dataset. Front Aging Neurosci 2022 Nov 10; 14:1036588*

45 *Suppa A, Costantini G, Asci F, Di Leo P, Al-Wardat MS, Di Lazzaro G, Scalise S, Pisani A, Saggio G. Voice in Parkinson's disease: a machine learning study. Front Neurol 2022 Feb 15; 13:831428*

46. *Song J, Lee JH, Choi J, Suh MK, Chung MJ, Kim YH, Park J, Choo SH, Son JH, Lee DY, Ahn JH, Youn J, Kim K, Cho JW. Detection and differentiation of ataxic and hypokinetic dysarthria in cerebellar ataxia and parkinsonian disorders via wave splitting and integrating neural networks. PLoS One 2022 Jun 3; 17(6):e0268337*

47. *Motin MA, Pah ND, Raghav S, Kumar DK. Parkinson’s disease detection using smartphone recorded phonemes in real world conditions. IEEE Access 2022; 10:97600-9*

48. *Maskeliūnas R, Damaševičius R, Kulikajevas A, Padervinskis E, Pribuišis K, Uloza V. A hybrid u-lossian deep learning network for screening and evaluating Parkinson’s disease. Appl Sci 2022 Nov 15; 12(22):11601*

49. *Lim WS, Chiu SI, Wu MC, Tsai SF, Wang PH, Lin KP, Chen Y, Peng P, Chen Y, Jang JR, Lin C. An integrated biometric voice and facial features for early detection of Parkinson's disease. NPJ Parkinsons Dis 2022 Oct 29; 8(1):145*

50. *Laganas C, Iakovakis D, Hadjidimitriou S, Charisis V, Dias SB, Bostantzopoulou S, Katsarou Z, Klingelhoefer L, Reichmann H, Trivedi D, Chaudhuri KR, Hadjileontiadis LJ. Parkinson's disease detection based on running speech data from phone calls. IEEE Trans Biomed Eng 2022 May; 69(5):1573-84*

51. *Fayad R, Hajj-Hassan M, Costantini G, Zarazadeh Z, Errico V, Pisani A. Vocal test analysis for assessing Parkinson's disease at early stage. In: Proceedings of the 6th International Conference on Advances in Biomedical Engineering. 2021. Presented at: ICABME '14; October 7-9, 2021; Werdanyeh, Lebanon. p. 171-4*

52. *Rahman W, Lee S, Islam MS, Antony VN, Ratnu H, Ali MR, Mamun AA, Wagner E, Jensen-Roberts S, Waddell E, Myers T, Pawlik M, Soto J, Coffey M, Sarkar A, Schneider R, Tarolli C, Lizarraga K, Adams J, Little MA, Dorsey ER, Hoque E. Detecting Parkinson disease using a web-based speech task: observational study. J Med Internet Res 2021 Oct 19; 23(10):e26305*.

53. *Cordella F, Paffi A, Pallotti A. Classification-based screening of Parkinson’s disease patients through voice signal. In: Proceedings of the 2021 IEEE International Symposium on Medical Measurements and Applications. 2021. Presented at: MeMeA '21; June 23-25, 2021; Lausanne, Switzerland. p. 1-6*.

54. *Vasquez-Correa JC, Arias-Vergara T, Klumpp P, Perez-Toro PA, Orozco-Arroyave JR, Nöth E. End-2-end modeling of speech and gait from patients with Parkinson’s disease: comparison between high quality vs. smartphone data. In: Proceedings of the 2021 IEEE International Conference on Acoustics, Speech and Signal Processing. 2021. Presented at: ICASSP '21; June 6-11, 2021; Toronto, ON. p. 7298-302*

55. *Majda-Zdancewicz, E.Potulska-Chromik, A.Jakubowski, J.Nojszewska, M.Kostera-Pruszczyk A. Deep learning vs feature engineering in the assessment of voice signals for diagnosis in Parkinson's disease. Bulletin of the Polish Academy of Sciences: Technical Sciences. 2021;69(3). doi: 10.24425/bpasts.2021.137347*

56. *Quan C, Ren K, Luo Z. A deep learning based method for Parkinson’s disease detection using dynamic features of speech. IEEE Access 2021; 9:10239-52*.

57. *Amato F, Borzi L, Olmo G, Artusi CA, Imbalzano G, Lopiano L. Speech impairment in Parkinson’s disease: acoustic analysis of unvoiced consonants in Italian native speakers. IEEE Access 2021; 9:166370-81*.

58. *Tandjung MD, Wu JC, Wang JC, Li YH. An implementation of FastAI tabular learner model for Parkinson’s disease identification. In: Proceedings of the 9th International Conference on Orange Technology. 2021. Presented at: ICOT '21; December 16-17, 2021; Tainan, Taiwan. p. 16-7*.

59. *Jeancolas L, Petrovska-Delacrétaz D, Mangone G, Benkelfat BE, Corvol JC, Vidailhet M, Lehéricy S, Benali H. X-vectors: new quantitative biomarkers for early Parkinson’s disease detection from speech. Front Neuroinform 2021 Feb 19; 15:578369*.

60. *Goyal J, Khandnor P, Aseri TC. A hybrid approach for Parkinson’s disease diagnosis with resonance and time-frequency based features from speech signals. Expert Syst Appl 2021 Nov; 182:115283*.

61. *Carrón J, Campos-Roca Y, Madruga M, Pérez CJ. A mobile-assisted voice condition analysis system for Parkinson's disease: assessment of usability conditions. Biomed Eng Online 2021 Nov 21; 20(1):114*.

62. *Ali L, He Z, Cao W, Rauf HT, Imrana Y, Bin Heyat MB. MMDD-ensemble: a multimodal data-driven ensemble approach for Parkinson’s disease detection. Front Neurosci 2021 Nov 1; 15:754058*.

63. *Zhang L, Qu Y, Jin B, Jing L, Gao Z, Liang Z. An intelligent mobile-enabled system for diagnosing Parkinson disease: development and validation of a speech impairment detection system. JMIR Med Inform 2020 Sept 16; 8(9):e18689*.

64. *Altay EV, Alatas B. Association analysis of Parkinson disease with vocal change characteristics using multi-objective metaheuristic optimization. Med Hypotheses 2020 Aug; 141:109722*.

65. *Vasquez-Correa JC, Arias-Vergara T, Orozco-Arroyave JR, Eskofier B, Klucken J, Noth E. Multimodal assessment of Parkinson's disease: a deep learning approach. IEEE J Biomed Health Inform 2019 Jul; 23(4):1618-30*.

66. *Camnos-Roca Y, Calle-Alonso F, Perez CJ, Naranjo L. Computational diagnosis of Parkinson’s disease from speech based on regularization methods. In: Proceedings of the 26th European Signal Processing Conference. 2018. Presented at: EUSIPCO '18; September 3-7, 2018; Rome, Italy. p. 1127-31*.

67. *Montaña D, Campos-Roca Y, Pérez CJ. A Diadochokinesis-based expert system considering articulatory features of plosive consonants for early detection of Parkinson's disease. Comput Methods Programs Biomed 2018 Feb; 154:89-97*.

68. *Vaiciukynas E, Verikas A, Gelzinis A, Bacauskiene M. Detecting Parkinson's disease from sustained phonation and speech signals. PLoS One 2017 Oct 5; 12(10):e0185613*.

69. *Sztahó D, Tulics MG, Vicsi K, Valálik I. Automatic estimation of severity of Parkinson's disease based on speech rhythm related features. In: Proceedings of the 8th IEEE International Conference on Cognitive Infocommunications. 2017. Presented at: CogInfoCom '17; September 11-14, 2017; Debrecen, Hungary. p. 11-6*.

70. *Orozco-Arroyave R, Arias-Londoño JD, Vargas-Bonilla JJ, Nöth E. Perceptual analysis of speech signals from people with Parkinson’s disease. In: Proceedings of the 5th International Work-Conference on the Interplay Between Natural and Artificial Computation & Natural and Artificial Models in Computation and Biology. 2013. Presented at: IWINAC '13; June 10-14, 2013; Mallorca, Spain. p. 201-11*.

71. *Sakar BE, Isenkul ME, Sakar CO, Sertbas A, Gurgen F, Delil S, Apaydin H, Kursun O. Collection and analysis of a Parkinson speech dataset with multiple types of sound recordings. IEEE J Biomed Health Inform 2013 Jul; 17(4):828-34*

72. *Viswanathan R, Bingham A, Raghav S, Arjunan SP, Jelfs B, Kempster P, Kumar DK. Normalized Mutual Information of phonetic sound to distinguish the speech of Parkinson's disease. Annu Int Conf IEEE Eng Med Biol Soc 2019 Jul; 2019:3523-6*

73. *Zhang H, Yan N, Wang L, Ng ML. Energy distribution analysis and nonlinear dynamical analysis of phonation in patients with Parkinson's disease. In: Proceedings of the 2017 Asia-Pacific Signal and Information Processing Association Annual Summit and Conference. 2017. Presented at: APSIPA-ASC '17; December 12-15, 2017; Kuala Lumpur, Malaysia. p. 630-5*.

74. *Vizza P, Tradigo G, Mirarchi D, Bossio RB, Lombardo N, Arabia G, Quattrone A, Veltri P. Methodologies of speech analysis for neurodegenerative diseases evaluation. Int J Med Inform 2019 Feb; 122:45-54*.

75. *Viswanathan R, Arjunan SP. Estimation of severity in Parkinson’s disease using acoustic features of phonatory tasks. IETE J Res 2021 Nov 22; 69(9):6292-303*.

76. *Das B, Daoudi K, Klempir J, Rusz J. Towards disease-specific speech markers for differential diagnosis in Parkinsonism. In: Proceedings of the 2019 IEEE International Conference on Acoustics, Speech and Signal Processing. 2019. Presented at: ICASSP '19; May 12-17, 2019; Brighton, UK. p. 2019-2019*.

77. *Li G, Daoudi K, Klempir J, Rusz J. Linear classification in speech-based objective differential diagnosis of parkinsonism. In: Proceedings of the 2018 IEEE International Conference on Acoustics, Speech and Signal Processing. 2018. Presented at: ICASSP '18; April 15-20, 2018; Calgary, AB. p. 15-20*

78. *Hemmerling D, Wojcik-Pedziwiatr M. Prediction and estimation of Parkinson's disease severity based on voice signal. J Voice 2022 May; 36(3):439.e9-20*

79. *Tunc HC, Sakar CO, Apaydin H, Serbes G, Gunduz A, Tutuncu M, Gurgen F. Estimation of Parkinson's disease severity using speech features and extreme gradient boosting. Med Biol Eng Comput 2020 Nov 10; 58(11):2757-73*.

80. *Vandana VP, Darshini JK, Vikram VH, Nitish K, Kumar PP, Ravi Y. Speech characteristics of patients with Parkinson's disease-does dopaminergic medications have a role?. J Neurosci Rural Pract 2021 Oct; 12(4):673-9*

81. *Jain A, Abedinpour K, Polat O, Çalışkan MM, Asaei A, Pfister FM, Fietzek UM, Cernak M. Voice analysis to differentiate the dopaminergic response in people with Parkinson’s disease. Front Hum Neurosci 2021 May 31; 15:667997*

82. *Gaballah A, Parsa V, Andreetta M, Adams S. Assessment of amplified parkinsonian speech quality using deep learning. In: Proceedings of the 2018 IEEE Canadian Conference on Electrical & Computer Engineering. 2018. Presented at: CCECE '18; May 13-16, 2018; Quebec, QC. p. 1-4*

83. *Gaballah A, Parsa V, Andreetta M, Adams S. Objective and subjective speech quality assessment of amplification devices for patients with Parkinson’s disease. IEEE Trans Neural Syst Rehabil Eng 2019 Jun; 27(6):1226-35*

86. *Shimoda A, Li Y, Hayashi H, Kondo N. Dementia risks identified by vocal features via telephone conversations: a novel machine learning prediction model. PLoS One 2021 Jul 14; 16(7):e0253988*.

87. *Toth L, Hoffmann I, Gosztolya G, Vincze V, Szatloczki G, Banreti Z, Pakaski M, Kalman J. A speech recognition-based solution for the automatic detection of mild cognitive impairment from spontaneous speech. Curr Alzheimer Res 2018; 15(2):130-8*.

88. *Themistocleous C, Eckerström M, Kokkinakis D. Identification of mild cognitive impairment from speech in Swedish using deep sequential neural networks. Front Neurol 2018 Nov 15; 9:975*.

89. *Nagumo R, Zhang Y, Ogawa Y, Hosokawa M, Abe K, Ukeda T, Sumi S, Kurita S, Nakakubo S, Lee S, Doi T, Shimada H. Automatic detection of cognitive impairments through acoustic analysis of speech. Curr Alzheimer Res 2020 Mar 20; 17(1):60-8*.

90. *Munthuli A, Vongsurakrai S, Anansiripinyo T, Ellermann V, Sroykhumpa K, Onsuwan C, Chutichetpong P, Hemrungrojn S, Kosawat K, Tantibundhit C. Thammasat-NECTEC-Chula's Thai language and cognition assessment (TLCA): the Thai Alzheimer's and mild cognitive impairment screening test. Annu Int Conf IEEE Eng Med Biol Soc 2021 Nov; 2021:690-4*.

91. *Mirzaei S, El Yacoubi M, Garcia-Salicetti S, Boudy J, Kahindo C, Cristancho-Lacroix V, Kerhervé H, Rigaud A. Two-stage feature selection of voice parameters for early Alzheimer's disease prediction. IRBM 2018 Dec; 39(6):430-5*.

92. *König A, Satt A, Sorin A, Hoory R, Toledo-Ronen O, Derreumaux A, Manera V, Verhey F, Aalten P, Robert PH, David R. Automatic speech analysis for the assessment of patients with predementia and Alzheimer's disease. Alzheimers Dement (Amst) 2015 Mar; 1(1):112-24*.

93. *Bertini F, Allevi D, Lutero G, Montesi D, Calzà L. Automatic speech classifier for mild cognitive impairment and early dementia. ACM Trans Comput Healthcare 2021 Oct 15; 3(1):1-11*.

94. *Yamada Y, Shinkawa K, Nemoto M, Ota M, Nemoto K, Arai T. Speech and language characteristics differentiate Alzheimer's disease and dementia with Lewy bodies. Alzheimers Dement (Amst) 2022; 14(1):e12364*.

95. *Sumali B, Mitsukura Y, Liang KC, Yoshimura M, Kitazawa M, Takamiya A, Fujita T, Mimura M, Kishimoto T. Speech quality feature analysis for classification of depression and dementia patients. Sensors (Basel) 2020 Jun 26; 20(12):3599*.

96. A*l-Hameed S, Benaissa M, Christensen H, Mirheidari B, Blackburn D, Reuber M. A new diagnostic approach for the identification of patients with neurodegenerative cognitive complaints. PLoS One 2019; 14(5):e0217388*.

97. *König A, Mallick E, Tröger J, Linz N, Zeghari R, Manera V, Robert P. Measuring neuropsychiatric symptoms in patients with early cognitive decline using speech analysis. Eur Psychiatry 2021 Oct 13; 64(1):e64*.

99. *Fazeli M, Moradi N, Soltani M, Naderifar E, Majdinasab N, Latifi SM, Dastoorpour M. Dysphonia characteristics and vowel impairment in relation to neurological status in patients with multiple sclerosis. J Voice 2020 May; 34(3):364-70*.

100. *Fazeli M, Moradi N, Soltani M, Naderifar E, Majdinasab N, Latifi SM. Comparison of dysphonia severity index and its parameters among individuals with multiple sclerosis and healthy subjects. Shiraz E Med J 2018 Jun 12; 19(7):e64857*.

101. *Svoboda E, Bořil T, Rusz J, Tykalová T, Horáková D, Guttmann C, Blagoev K, Hatabu H, Valtchinov V. Assessing clinical utility of machine learning and artificial intelligence approaches to analyze speech recordings in multiple sclerosis: a pilot study. Comput Biol Med 2022 Sept; 148:105853*

102. *Gosztolya G, Tóth L, Svindt V, Bóna J, Hoffmann I. Using acoustic deep neural network embeddings to detect multiple sclerosis from speech. In: Proceedings of the 2022 IEEE International Conference on Acoustics, Speech and Signal Processing. 2022. Presented at: ICASSP '22; May 23-27, 2022; Singapore, Singapore. p. 6927-31*.

103. *Vizza P, Mirarchi D, Tradigo G, Redavide M, Bossio RB, Veltri P. Vocal signal analysis in patients affected by multiple sclerosis. Procedia Comput Sci 2017; 108:1205-14*.

105. *Tena A, Claria F, Solsona F, Meister E, Povedano M. Detection of bulbar involvement in patients with amyotrophic lateral sclerosis by machine learning voice analysis: diagnostic decision support development study. JMIR Med Inform 2021 Mar 10; 9(3):e21331*.

106. *Illa A, Patel D, Yamini B, ss M, Shivashankar N, Veeramani P. Comparison of speech tasks for automatic classification of patients with amyotrophic lateral sclerosis and healthy subjects. In: Proceedings of the 2018 IEEE International Conference on Acoustics, Speech and Signal Processing. 2018. Presented at: ICASSP '18; April 15-20, 2018; Calgary, AB. p. 6014-8*.

107. *Likhachov D, Vashkevich M, Azarov E, Malhina K, Rushkevich Y. A mobile application for detection of amyotrophic lateral sclerosis via voice analysis. In: Proceedings of the 23rd International Conference on Speech and Computer. 2021. Presented at: SPECOM '21; September 27-30, 2021; St. Petersburg, Russia. p. 372-83*.

108. *Mallela J, Illa AS, N SB, Udupa S, Belur Y, Atchayaram N, Yadav R, Reddy P, Gope D, Ghosh PK. Voice based classification of patients with amyotrophic lateral sclerosis, Parkinson’s disease and healthy controls with CNN-LSTM using transfer learning. In: Proceedings of the 2020 IEEE International Conference on Acoustics, Speech and Signal Processing. 2020. Presented at: ICASSP '20; May 4-8, 2020; Barcelona, Spain. p. 6784-8*.

110. *Daudet L, Yadav N, Perez M, Poellabauer C, Schneider S, Huebner A. Portable mTBI assessment using temporal and frequency analysis of speech. IEEE J Biomed Health Inform 2017 Mar; 21(2):496-506*.

111. *Falcone M, Yadav N, Poellabauer C, Flynn P. Using isolated vowel sounds for classification of mild traumatic brain injury. In: Proceedings of the 2013 IEEE International Conference on Acoustics, Speech and Signal Processing. 2013. Presented at: ICASSP '13; May 16-31, 2013; Vancouver, BC. p. 26-31*.

112. *Wall C, Powell D, Young F, Zynda AJ, Stuart S, Covassin T, Godfrey A. A deep learning-based approach to diagnose mild traumatic brain injury using audio classification. PLoS One 2022; 17(9):e0274395*.

113. *Riad R, Lunven M, Titeux H, Cao XN, Hamet Bagnou J, Lemoine L, Montillot J, Sliwinski A, Youssov K, Cleret de Langavant L, Dupoux E, Bachoud-Lévi AC. Predicting clinical scores in Huntington's disease: a lightweight speech test. J Neurol 2022 Sept 14; 269(9):5008-21*.

114. *Gallezot C, Riad R, Titeux H, Lemoine L, Montillot J, Sliwinski A, Bagnou JH, Cao XN, Youssov K, Dupoux E, Bachoud Levi A. Emotion expression through spoken language in Huntington disease. Cortex 2022 Oct; 155:150-61*.

116. *MacFarlane H, Salem AC, Chen L, Asgari M, Fombonne E. Combining voice and language features improves automated autism detection. Autism Res 2022 Jul 23; 15(7):1288-300*.

117. *Eni M, Dinstein I, Ilan M, Menashe I, Meiri G, Zigel Y. Estimating autism severity in young children from speech signals using a deep neural network. IEEE Access 2020; 8:139489-500*.

119. *Suppa A, Asci F, Saggio G, Di Leo P, Zarezadeh Z, Ferrazzano G, Ruoppolo G, Berardelli A, Costantini G. Voice analysis with machine learning: one step closer to an objective diagnosis of essential tremor. Mov Disord 2021 Jun 02; 36(6):1401-10*.

120. *König A, Linz N, Zeghari R, Klinge X, Tröger J, Alexandersson J, Robert P. Detecting apathy in older adults with cognitive disorders using automatic speech analysis. J Alzheimers Dis 2019; 69(4):1183-93*.

122. *Aggarwal G, Sharma NV, Kavita, Sinha A. Fisher discriminant ratio based classification of intellectual disability using acoustic features. In: Proceedings of the 2nd International Conference on International Conference. 2020. Presented at: CNC '20; December 29-31, 2020; Gwalior, India. p. 301-11*.

123. *Lauraitis A, Maskeliunas R, Damasevicius R, Krilavicius T. Detection of speech impairments using cepstrum, auditory spectrogram and wavelet time scattering domain features. IEEE Access 2020; 8:96162-72*.
